# Structure and Sequence of the Sex Determining Locus in Two Wild Populations of Nile Tilapia

**DOI:** 10.3390/genes11091017

**Published:** 2020-08-29

**Authors:** Cécile Triay, Matthew A. Conte, Jean-François Baroiller, Etienne Bezault, Frances E. Clark, David J. Penman, Thomas D. Kocher, Helena D’Cotta

**Affiliations:** 1 UMR-Institut des Sciences de l’Evolution de Montpellier, Centre National de la Recherche Scientifique, Institut de Recherche pour le Développement, Ecole Pratique des Hautes Etudes, University of Montpellier, 34090 Montpellier, France; cecile.triay@cirad.fr (C.T.); baroiller@cirad.fr (J.-F.B.); 2 UMR-Institut des Sciences de l’Evolution de Montpellier, Centre de Coopération Internationale en Recherche Agronomique pour le Développement, Campus Int. Baillarguet, 34398 Montpellier, France; 3 Department of Biology, University of Maryland, College Park, MD 20742, USA; mconte@umd.edu (M.A.C.); betsy.clark@nih.gov (F.E.C.); tdk@umd.edu (T.D.K.); 4 UMR BOREA, CNRS-7208/MNHN/UPMC/IRD-207/UCN/UA, Université des Antilles, 97159 Guadeloupe, France; etienne.bezault@univ-antilles.fr; 5 Institute of Aquaculture, School of Natural Sciences, University of Stirling, Stirling, Scotland FK9 4LA, UK; davidpenman59@gmail.com

**Keywords:** sex-determinant, Y-chromosome, amh, male duplication, Nile tilapia

## Abstract

In domesticated strains of the Nile tilapia, phenotypic sex has been linked to genetic variants on linkage groups 1, 20 and 23. This diversity of sex-loci might reflect a naturally polymorphic sex determination system in Nile tilapia, or it might be an artefact arising from the process of domestication. Here, we searched for sex-determiners in wild populations from Kpandu, Lake Volta (Ghana-West Africa), and from Lake Koka (Ethiopia-East Africa) that have not been subjected to any genetic manipulation. We analysed lab-reared families using double-digest Restriction Associated DNA sequencing (ddRAD) and analysed wild-caught males and females with pooled whole-genome sequencing (WGS). Strong sex-linked signals were found on LG23 in both populations, and sex-linked signals with LG3 were observed in Kpandu samples. WGS uncovered blocks of high sequence coverage, suggesting the presence of B chromosomes. We confirmed the existence of a tandem *amh* duplication in LG23 in both populations and determined its breakpoints between the *oaz1* and *dot1l* genes. We found two common deletions of ~5 kb in males and confirmed the presence of both *amhY* and *amh∆Y* genes. Males from Lake Koka lack both the previously reported 234 bp deletion and the 5 bp frameshift-insertion that creates a premature stop codon in *amh∆Y*.

## 1. Introduction

The male heterogametic (XY) system of sex determination in mammals, and the female heterogametic (ZW) system in birds, are evolutionarily conserved in most species of their respective clades [1,2]. Furthermore, the master sex-determining gene on the Y chromosome, *sry*, is almost universal across the mammalian class [3]. The sex chromosomes of most mammals and birds are highly differentiated due to the suppression of recombination, and the subsequent accumulation of deleterious mutations which has resulted in the degeneration of the Y (or W) chromosomes (reviewed by Bachtrog [4]). Fish in contrast, usually have homomorphic sex chromosomes, so the sex of an individual typically cannot be distinguished by karyotyping. Genetic sex determination (GSD) has been established for many fish species by progeny testing, sex-reversal treatments and by the association of genetic markers with phenotypic sex (see reviews of Baroiller et al. [5]; Devlin and Nagahama [6]). These studies have demonstrated a wide range of GSD mechanisms, including both mono- and polygenic systems. Sex determiners have been described on undifferentiated autosomes and well-differentiated sex chromosomes, and in some cases interact with environmental factors to determine phenotypic sex [7].

Frequent replacement of fish sex-determining systems is inferred from the fact that both male XY and female ZW heterogametic systems can be found between and even within fish lineages [8,9]. The existence of multiple sex chromosomes (e.g., *Xiphophorus*) or polygenic systems (e.g., *Dicentrarchus labrax*) within species also suggest the dynamic evolution of fish sex chromosomes [10,11,12]. The diversity and rapid turnover of fish sex chromosomes have been made more apparent with the identification of sex-determination genes. The master sex-determining gene of the medaka *Oryzias latipes* evolved from a duplication of *dmrt1* [13,14]. In another *Oryzias* species, the sex-determinant is a Y-specific copy of the *gsdf* gene [15]. The sex-determining gene in the Patagonian pejerrey *Odontesthes hatcheri* is a duplication of the anti-Müllerian hormone *amh* [16,17]. *Amh* has also been duplicated to become the sex-determining gene in the Northern pike *Esox lucius* [18]. A male-specific allele of its receptor *amhr2* was identified as the sex-determinant in *Takifugu* species [19]. In contrast, the master sex-determination gene *sdy* of salmonids arose from the duplication of an immune-related gene [20].

African cichlids, particularly those that have undergone adaptive radiation in the large East African Lakes, show an extraordinary diversity of sex chromosomes. Studies of cichlids from Lake Tanganyika and Lake Malawi have identified more than twelve different sex-determining systems that have been mapped to at least ten distinct chromosomes, including ZW systems on LG5 and LG7 and XY systems mapping to LG7, LG19 and LG20 [9,21,22,23]. A complex scenario has been found for various populations of *Astatotilapia burtoni*, including an XYW system mapped to LG13, an XY system on LG18 and an XY system on a fusion comprising LG5 and LG14 [24,25]. In addition, some species from Lake Malawi possess female-specific B chromosomes that carry a female sex determiner, that is epistatically dominant to the LG7 XY system [26,27].

Due to economic interest in producing fast-growing all-male stocks for aquaculture, numerous studies have examined the sex-determining system in the Nile tilapia (*Oreochromis niloticus*) [28]. These studies have revealed a complex sex-determining system, involving both genetic and environmental factors [12,29,30,31]. Many of these studies have been performed on domesticated or laboratory strains, which may have experienced several rounds of selection, introgressive hybridizations, and/or significant inbreeding [32,33].

Cytogenetic observations of the synaptonemal complex in Nile tilapia revealed that the terminal ends of the largest chromosome (LG3) remained unpaired in males, suggesting this was the sex chromosome [34]. However, the genetic mapping of families showed a sex linkage of markers on a smaller chromosome (LG1) [29,30,35]. In other strains of *O. niloticus* sex was linked to LG23 [36,37]. Genomic studies also showed that sex was associated with either LG1 [31,38,39,40] or LG23 [40,41,42,43,44] depending on the strain. The *amh* gene, on LG23, has since been identified as the sex-determining gene in a Japanese strain [43]. The Y has two tandem copies, *amhY* [43] and *amhΔY*, which has a premature stop codon due to an insertion [42,43], while the X chromosome has a single copy, here called *amhX*. Knock-out studies showed that *amhY* is necessary for determining maleness [43]. When not a sex determinant, the *amh* still intervenes in gonad development, in germ cell regulation and is even expressed in the brains of some teleost species (reviewed by Pfenning et al. [45]).

It is not clear to what extent these differences in sex-determination of Nile tilapia are due to natural diversity in the mechanisms of sex determination or due to processes of domestication (including introgressive hybridizations whether incidental or not). Domestication of zebrafish (*Danio rerio*) caused the loss of the strong sex determiner on chromosome 4 present in wild fish [46]. Studies of wild Nile tilapia from Lake Kou showed variability in the Y-linked *amh* sequences on LG23 and also suggested that another locus was also involved in sex determination [47]. The objective of the present study was to characterize the sex determiner(s) in wild populations of Nile tilapia that have not been subjected to any form of domestication or genetic manipulations. We investigated a population from West Africa (Ghana) and another from East Africa (Ethiopia), using two complementary genomic approaches to analyse lab-reared families as well as pools of wild-caught males and females.

## 2. Materials and Methods

### 2.1. Fish and DNA Samples

**Figure 1:**
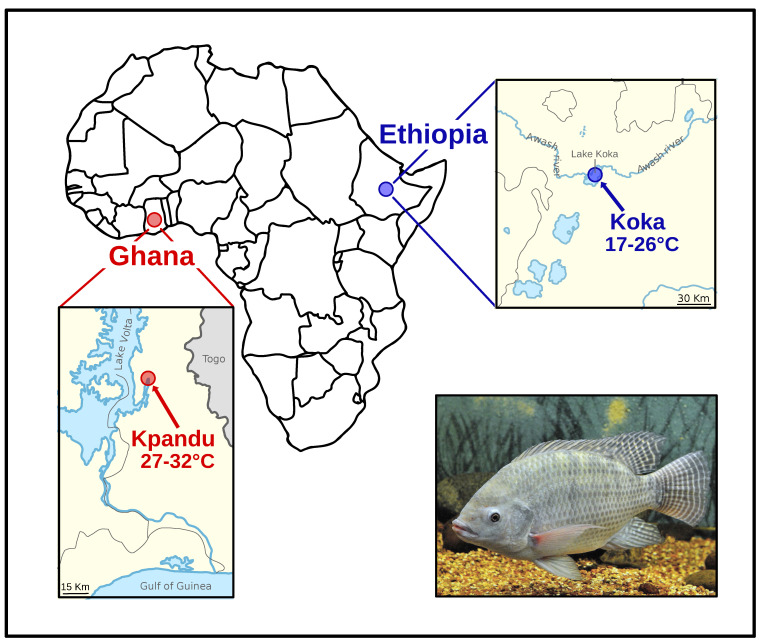
Map of Africa showing Ghana in West Africa with the location of the Kpandu population in Lake Volta (with medium to hot temperatures) and Lake Koka (a cold lake) located in the Ethiopian highlands of the East African Rift.

Nile tilapia samples originated from two wild populations caught in Lake Volta (Kpandu, Ghana) in 2002 and 2003 and Lake Koka (Ethiopia) in 2002. The fish from Kpandu belong to the subspecies *Oreochromis niloticus niloticus*, whereas those from Lake Koka are the subspecies *Oreochromis niloticus cancellatus*. They were sexually mature and could be phenotypically sexed by their genital papilla, as described in Bezault et al. (2007) [48]. The collection site near Kpandu is a dendritic expansion on the eastern side of Lake Volta in Ghana (Figure 1) where temperature varies throughout the year between 27 °C and 32 °C. Lake Koka is a reservoir of the Awash River in the Ethiopian Highlands of East Africa, where temperatures vary throughout the year between 21–26 °C and therefore it can be considered a “cold lake” (Figure 1). Wild individuals were transferred to the CIRAD experimental facilities in Montpellier (France), where they were progressively acclimated to a standard temperature of 27 °C ± 1 °C in a thermo-regulated re-circulating system and individually tagged. 

For the double-digest Restriction Associated DNA (ddRAD) experiment, we produced an F1 family from each population by crossing the wild-caught individuals in 240 L aquaria. The Kpandu Kp20 progeny was obtained by crossing male KpM17 to female KpF32. The Koka Kk-6 progeny was obtained by crossing male KkM39 and female KkF4 (Table 1). Fry were collected from the mother’s mouth at 3 days post-fertilisation (dpf), transferred to McDonald jars for incubation until 9 or 10 dpf, and then reared in 40 L aquaria at 27 ± 1 °C. At 90 dpf, fish were euthanised with 2-phenoxyethanol (1 mL/L) and 100 randomly sampled individuals per group were sexed by microscopic observation of the gonads [28] to determine the phenotypic sex and define the sex ratios of the progenies. Fin clips from the parents, and from 14 females and 15 males per family were collected and stored in 100% ethanol until DNA extraction. We deliberately selected families that gave balanced sex-ratios when reared at 27 °C. This minimized the effects of environmental or minor genetic factors that might bias the proportion of males. The Kpandu Kp-20 family showed a sex-ratio of 46% males at 27 °C which was not significantly different from the standard balanced (1:1) sex ratio (Chi-square test NS (Non Significant) *p* > 0.05) (Table 1). The Koka Kk-6 progeny also gave a balanced sex ratio of 55% males NS for *p* > 0.05) at 27 °C.

Fin clips for the Whole Genome Sequencing (WGS) were taken from the wild-caught individuals, consisting of 14 females and 20 males from Koka, and 27 females and 27 males from Kpandu (Table 1). To validate the sex chromosome markers we used fin clips from fish of known sexual genotype from the Manzala-Tihange strain (Belgium) kept at CIRAD. They were an XX female, an XY male and a YY supermale.

### 2.2. Ethics Statement

All animal and experimental procedures were performed at the CIRAD facilities in Montpellier in accordance with the French protocol N°2016101810463 and the author’s personal authorization for animal experimentation N° 35–15, both delivered by the French Government. Fish captures followed the laws and veterinary agreements of each country as stipulated in 2003.

### 2.3. DNA Extraction

Genomic DNA (gDNA) was extracted in 96 well plates from fin clips cut to 0.5 × 0.5 cm size, digested in 300 µL lysis solution containing 0.3 M NaCl, 50 mM Tris-base, 0.2 Mm EDTA, 0.2 mM EGTA, 0.356 mM spermidine, 0.256 mM spermine, 4.8% SDS and 10 µg proteinase K at 55 °C overnight. This was followed by an inactivation at 70 °C for 10 min and then by treatment with 6.6 µg RNAse for 1 h at 37 °C. After brief centrifugation, the clear supernatant was extracted with the King Fisher Flex robot using the Nucleomag 96 Tissue Kit (Macherey-Nagel, Dueren, Germany) following the commercial protocol. We only changed the buffer quantities, using 200 µL of MBE and MB4 per sample, 300 µL of MB5/sample, and 335 µL of MB2 + 25 µL of magnetic beads (diluted at 1/2 with Ambion water). The gDNA was eluted in 100 µL of MB6 (5 mM Tris) and after the residual magnetic beads were settled on a magnetic rack, a clear eluate was retained. DNA was quantified using the Qubit 2.0 fluorometer (Invitrogen, Carlsbad, CA, USA) dsDNA BR kit and the quality was checked with a Nanodrop spectrophotometer (ThermoFisher, Waltham, MA, USA) and on a 0.8% agarose gel. Finally, the gDNA was diluted to 50 ng/µL.

### 2.4. Sexual Genotyping with Amh X and Y Chromosome Markers

We genotyped the sex of the wild individuals (11 females and 15 males from Koka and 27 females and 27 males from Kpandu) using PCR amplification of four markers on LG23 shown to be specific for the X and/or the Y chromosome in domestic strains of Nile tilapia. These markers are located within the *amh* genes and adjacent promoter regions. Due to the high sequence homology of the 3 *amh* genes, the markers were designed to cover indels. The procedures were performed as described in Sissao et al. (2019) [47]. The first marker, *amhX*_+36_ (primers F1-GTTTGCAATAGTTAGGGTGCTGCTG; R- GGAAATGCAGCCATTCCTGAG) which amplifies a 1000 base pair (bp) fragment present on the promoter region of the *amh* gene located on the X chromosome [43]. The second marker, *amhΔY*_+5_ (primers F2-AAACCTCCTTCCTTTGTGAATGTC; R2-CTAGCGGCATCCACACTCCCTCAC) amplifies a 1500 bp fragment that corresponds to a 5 bp insertion in exon6 that is present in the *amhΔY* gene, located on the Y chromosome. This insertion causes a change in the reading frame resulting in a truncated protein [43]. The third marker, *amhΔY*_-233_ (primers F3-CGGTCCCAGTGACCTATGAG; R3-AAGTACACGTGGTGTATTGTAATTGA) gives two fragments; one of 1000 bp corresponding to either *amhX* and/or *amhY* genes, and another fragment at 767 bp, which corresponds to the *amhΔY* gene [42]. The fourth marker, *amhY*_-5608_ (primers F4-GAAAGGGGTGTTTTGGTGCTGGC; R4-ACCCAGGAAGCGTTTCATCTCA) amplifies two fragments: a 2414 bp band that specifically amplifies the *amhY* gene present on the Y chromosome, and a fragment of 8022 bp which specifically amplifies the *amhX* gene on the X chromosome [43]. We used this marker to detect the presence of the *amhY* gene in males, because the large *amhX* fragment does not amplify consistently. An XX female, XY male and YY male of the Manzala-Tihange (Belgium) strain were included as positive controls.

### 2.5. Double Digest RAD Sequencing

#### 2.5.1. Library Construction

The ddRAD library contained 64 individuals from two F1 families obtained from wild-caught crossings (Table 1) and was constructed following a protocol modified from Palaiokostas et al. (2015) [39], using 100 ng of gDNA per individual and a no-DNA control. Briefly, the gDNA was double digested for 3 h at 37 °C with 0.5 µL of *SbfI* and 0.5 µL of *SphI* (20 U/µL each, New England Biolabs (NEB), Ipswich, MA, USA) with 2 µL of 10X Cutsmart Buffer in a reaction volume of 20 µL. Restriction enzymes were then heat-inactivated at 65 °C for 15 min. Unique barcodes with specific combinations of P1 and P2 adaptors were used to identify all samples individually. We used 4 µL/sample of a 1:16 ratio consisting in 120 nM *SbfI* compatible P1 adaptor/1920 nM *SphI* compatible P2 adaptor with 0.5 µL of T4 Ligase (1000 U, 2000 U/µL, NEB), 0.3 µL rATP (100 nM, Promega, Madison, WI, USA), 1 µL 10X Cutsmart Buffer in a total volume of 6 µL/sample. Samples were incubated for 2 h at 22 °C. Subsequently, all samples were combined into a single pool, purified with the Nucleospin Gel & PCR clean-up kit (Macherel-Nagel, Dueren, Germany) and then eluted in 40 µL Elution Buffer (5 mM Tris). Size selection was performed by separating the eluate on a 1.2% agarose gel and excising fragments between 300 and 700 bp. These gel fragments were then purified with the QIAquick gel extraction kit (Qiagen, Dueren, Germany) and eluted in 3 × 30 µL Elution Buffer. The library was subsequently PCR amplified in 22 replicates, each containing 4 µL template, 1.35 µL of each PCR P1/P2 primers and 12.54 µL of Q5 Hot start HF 2X Master mix (NEB). The PCR program was: initial denaturation at 98 °C for 40 s, followed by 13 cycles of 10 s at 98 °C, 30 s at 65 °C, 45 s at 72 °C with a final elongation for 2 min at 72 °C. All PCR products were pooled and purified using the MinElute PCR purification kit (Qiagen). They were subsequently cleaned twice with AMPure XP magnetic beads (1× vol. followed by 0.7× vol.) to eliminate residual fragments under 300 bp and above 700 bp. The final library concentration was quantified with the HS dsDNA Qubit kit, and the quality and size estimated on a Bioanalyzer 2100 using a High Sensitivity DNA Chip (Agilent, Santa Clara, Californie, CA, USA), which confirmed that the majority of fragments were between 300–600 bp. The ddRAD library was sequenced at the GeT-PlaGe platform (Toulouse, Auzeville, France) on one lane of 150 bp paired-end reads on a HiSeq3000 (Illumina, San Diego, CA, USA).

#### 2.5.2. Data Processing

The data is accessible under the BioProject accession number PRJNA657179. The quality of the reads was checked using FastQC (0.11.7) [49]. Raw data were demultiplexed using the STACKS ‘process_radtags.pl’ script for paired-end reads [50]. The *SbfI-SphI* restriction enzymes and adapter sequences were specified and trimmed. Reads were then mapped to the *O. niloticus* reference genome O_niloticus_UMD_NMBU [51] using the BWA-MEM mapper (version 0.7.15) [52]. Output files from BWA-MEM (SAM files) were converted to BAM files, and then sorted and indexed with SAMtools (version 1.9) [53]. We then used STACKS (version 2.2) to complete the single nucleotide polymorphism (SNP) calling and population genetic statistics for each population separately [50]. STACKS implements a maximum likelihood statistical model algorithm to identify loci and call genotypes, thus reducing false polymorphic sites (e.g., due to sequencing errors). The STACKS script ‘ref_map.pl’ combines ‘pstacks’ to build loci, ‘cstacks’ to assemble a catalogue of loci, and ‘gstacks’ to match samples reads against the catalog. Finally, it runs ‘populations’ which computes the population genetics statistic AMOVA *F*_ST_ (derived from [54]) and Fisher’s Exact test between sexes at each nucleotide position. Relevant options passed to ‘populations’ were -M specifying a ‘population map’ with 2 groups, splitting samples according to their phenotypic sex; --smooth; --hwe; --fstats - f p_value --vcf. The output VCF files were filtered using VCF tools (0.1.14) [55] to keep sites with a maximum of 2 alleles (--max-alleles 2), and minimum read depth of 5 (--min-meanDP 5). We removed one phenotypic male of the Kpandu and one phenoypic female of the Koka offspring that had 2098 and 3261 times fewer reads than the mean of all individuals (--remove-indiv) in VCF files. Plain text table outputs were filtered using custom python and bash scripts. Only loci that had reads for at least half of the males and half of the females (per family) were kept.

### 2.6. Whole-Genome Sequencing

#### 2.6.1. Library Construction

Two libraries per population were produced for the WGS consisting of a pool of wild-caught females and a pool of wild-caught males. The two Kpandu libraries contained a pool of 27 females and a pool of 27 males, whilst the Koka libraries consisted of a pool of 14 females and a pool of 20 males (Table 1). Each pool contained an equal amount of individual gDNA, for a total of 2200 ng in 55 µL. Pooled DNA were sonicated using a Covaris S220 (Woburn, Massachusetts, MA, USA) to obtain 550 bp fragments. Libraries were subsequently prepared using the TruSeq DNA PCR-Free kit (Illumina, San Diego, CA, USA). Each pool was tagged with unique barcodes. Library quality and fragment size range were assessed using a High Sensitivity DNA Chip on the Bioanalyzer (Agilent, Santa Clara, CA, USA). Libraries were quantified by qPCR with the Library Quantification Kit (Takara Bio USA, Inc, Mountain View, CA, USA) on a Lightcycler 96 with the following program: initial denaturation of 2 min at 98 °C; 35 cycles of 10 s at 98 °C, 15 s at 60 °C and 45 s at 68 °C, finishing with a melting curve. The 4 libraries were sequenced on 2 lanes of a HiSeq 4000 (Illumina), using paired-end read lengths of 100 bp at the GenomEast Platform (Strasbourg, Illkirch, France).

#### 2.6.2. Data Processing

The data is accessible under the BioProject accession number PRJNA657179. The quality of the reads was checked using FastQC (version 0.11.7) [49]. Illumina TruSeq Adapters were trimmed using Trimmomatic (version 0.33) [56] for paired-end reads. The reads were mapped onto the XX female *O. niloticus* reference genome O_niloticus_UMD_NMBU using BWA mem mapper (version 0.7.15) [52]. SAM files were then converted to BAM files and sorted using SAMtools (version 1.9) [53]. PCR duplicates were identified using GATK MarkDuplicates (version 2.1.0) [57]. Files were subsequently indexed using Samtools index, and mpileup files were generated using the Samtools mpileup program. The output mpileup files were converted to sync files from Popoolation2 [58] with the ‘mpileup2sync.pl’ script and used as input for the next step. Fisher’s Exact Test were computed for both populations using popoolation2, designed for comparing allele frequencies between pooled sequencing data. *F*_ST_ was computed using the SEX_SNP_Finder_GA.pl pipeline [38] to identify SNPs at intermediate frequencies in males and fixed (or nearly fixed) in females. This was achieved by looking at both XY and ZW patterns for each population independently. The relevant parameters used were the following: --fixed_threshold = 0.9; --minimum_polymorphic_frequency = 0.3; --maximum_polymorphic_frequency = 0.7; --minimum_read_depth = 10; --maximum_read_depth = 100; --minimum_read_count = 2; --sex_SNP_finder_window_size = 10000. Structural variants were called using DELLY (0.8.1) [59], which focuses on paired-ends, split-reads, and read-depth to predict and discover genomic rearrangement. Small variants in the whole genome data were then called using FreeBayes (1.2.0) [60], a haplotype-based variant detector designed for small indels and polymorphisms. A filter was set for each pool independently to keep only variants for which the number of occurrences was at least 5% of the overall coverage. All graphs were made using R [61] with the qqman package for the Manhattan plot [62].

## 3. Results

### 3.1. Genotyping with Amh X and Y Chromosome Markers

**Figure 2:**
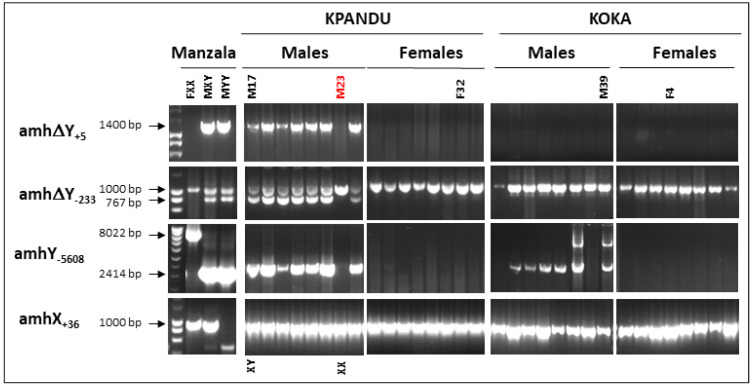
*Amh* genotyping of the Kpandu and Koka breeders used in ddRAD and a subset of individuals used in the whole-genome sequencing (WGS) pools. The complete genotyping of all individuals is shown in Figures S1 and S2.

The genotypic sex of the parents of the two families, and the other wild-caught fish from the Koka and Kpandu populations, was predicted by using four *amh* markers that have distinguished the X and Y chromosomes in the domesticated Manzala and Swansea strains (Israel) [42], the Japanese strain [43], and the Manzala-Tihange strain (Belgium) [47]. The presence of the Y chromosome in the majority of Kpandu males was proven with the successful amplification of the two *amhΔY* markers as well as the *amhY* marker. Indeed we amplified the 1400 bp band of the *amhΔY***_+5_** marker and the 767 bp fragment of the *amhΔY*_-233_ marker in the Kpandu males, like the control Manzala-Tihange males that possess the LG23 Y Chromosome (Figure 2). These results indicate the presence of the *amhΔY* gene and that these males, including the KPM17 sire, possess a Y chromosome and consequently are XY individuals. We found three Kpandu males without these bands suggesting they were XX males (Table 2; Figure 2 shows one of these males “M23”). All Kpandu females, including the KpF32 dam, were negative for these bands, indicating they were all XX females. None of the Koka males (or females) amplified the two *amhΔY* gene markers.

The presence of the *amhY* gene was confirmed using the *amhY*_-5608_ marker which gives a 2414 bp fragment belonging to the *amhY* and an 8022 bp fragment for *amhX* [43]. We used this marker to verify the presence of *amhY*, but the 8 kb *amhX* band amplifies poorly. We saw only the X fragment in the Manzala-Tihange XX female used as PCR control (Figure 2). We amplified the *amhY* fragment in all Kpandu males except the three XX males and did not amplify this fragment in Kpandu females. Weak amplification of the *amhY* fragment was observed in some Koka males, but not in any Koka females. All of the fish from Koka, Kpandu and the Manzala-Tihange strain (excluding the Manzala-Tihange YY male) amplified the 1000 bp *amhX*_+36_ marker and the X-specific 1000 bp band from the *amhΔY*_-233_ marker.

### 3.2. Read Quality and Mapping

#### 3.2.1. ddRAD Sequencing Libraries

We obtained a total of ~221 million clean reads for the Kpandu family after removing ambiguous RAD barcodes, orphaned paired-ends and low-quality reads. From these reads, an average of 94.9% per sample mapped properly paired to the *O. niloticus* reference genome (corresponding to an average of 7.1 million reads per individual). A total of 145,258 loci were built in STACKS, with an average insert length of 336.2 bp, and a mean of 7.6 males and 7.0 females mapped per locus. For the Koka family, a total of ~161 million clean reads were obtained, of which an average of 95.3% per sample mapped properly paired on the *O. niloticus* reference genome (corresponding to an average of 5.2 million reads per individual). This allowed us to detect 129,388 loci with a mean insert length of 329.3 bp. An average of 8.2 males and 7.4 females were mapped per locus. The number of reads per tag and other demultiplexing statistics for each sample are reported in Table S1.

#### 3.2.2. Whole-Genome Sequencing Libraries

We performed a pooled WGS strategy on the wild-caught samples of males and females (Table 1). We obtained ~115 million paired reads for the Koka female pool, ~93 million for Koka males, ~104 million for Kpandu females and ~226 million for Kpandu males. After trimming, we retained between 98 and 99% of the reads from each library. The mapping success onto the *O. niloticus* reference genome was over 94% for each library, with a least 83% correct paired reads. The average coverage obtained for Kpandu males was 39X, which is more than twice the average coverage for the other pools (Kpandu females: 18X, Koka females: 19X, Koka males: 16X).

### 3.3. Sex-Associated Loci Identified by ddRAD

#### 3.3.1. Sex-Associated Signal is Observed on LG23 and LG3

**Figure 3:**
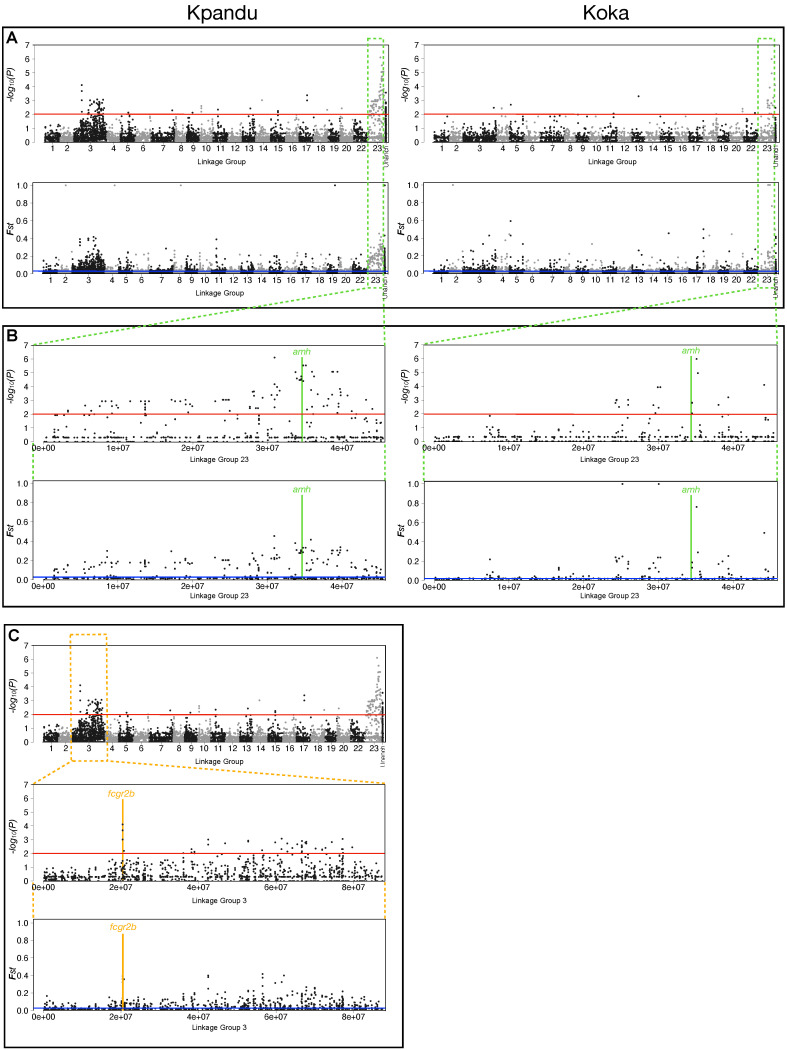
Manhattan plots of log10-transformed *p*-values from Fisher’s Exact Test (FET) and AMOVA *F_ST_* of the association of single nucleotide polymorphisms (SNPs) with phenotypic sex. (**A**) Plots for the Kpandu and the Koka families for all LGs. Unanchored contigs/scaffolds are grouped under the label “Unanch”. (**B**) Plots for the LG23 region showing SNP sites that were associated with sex. Most of the stronger sex-signals are found between 24 and 40 Mb. (**C**) Plots for Kpandu family with a close up of the LG3 region showing a very localised signal at 20 Mb due to a RAD locus in the *fcgr2b* gene. The rest of the signal observed on LG3 comes from the “long arm” of the chromosome known to carry repeated elements. Red lines correspond to *p*-values of 0.01 in the FET plots while blue lines correspond to mean AMOVA *F_ST_* between males and females of each family.

For the Kpandu family, we identified 14,073 polymorphic sites in phenotypic males and females located in 6437 RAD loci. For the Koka family, we identified 10,314 polymorphic sites in phenotypic males and females located in 4890 RAD loci. In both families, we found a significant peak of differentiation between phenotypic males and females on LG23, with 29 SNPs associated with sex for Koka and 125 SNPs for Kpandu (Fisher’s Exact Test *p*-value < 0.01) (Figure 3A and Table S2). The Manhattan plots of the *F*_ST_ values are similar for the two families. For the Kpandu family, 220 sites had a higher *F*_ST_ than the mean *F*_ST_ of 0.028. For the Koka family, there were 151 sites above the mean *F*_ST_ of 0.024.

The region of LG23 between 24 Mb and 40 Mb contains the majority of the signal for both families (Figure 3B). The Kpandu family showed a strong sex association of markers in this region. This region contained 62% of the significant sites, although we can observe significant sex-associated SNPs all along this linkage group. In the Koka family, sex was even more strongly and specifically associated with this region, which included 97% of the significantly differentiated sites. The overall lower number of significantly differentiated sites in the Koka family might be due to the lower level of sequence coverage (Table S1).

In the Kpandu family, we also obtained a sex-associated signal on LG3 (Figure 3C and Table S2). The highest signals on LG3 were located at 20Mb according to Fisher’s exact test. It corresponds to two RAD loci, one localised in a non-coding region and the other is in the “Fc fragment of IgG receptor IIb” gene (*fcgr2b*) (Table S3 and Figure 3C). More than 40 other significant sex-patterned SNPs on LG3 (Fisher’s Exact Test (FET) *p*-value < 0.001) occur in a region spanning ~40 Mb between 40 to 80 Mb (Table S3 and Figure 3C).

#### 3.3.2. Inherited Patterns of Offspring on LG23 Confirm a Male Heterogametic System

**Figure 4:**
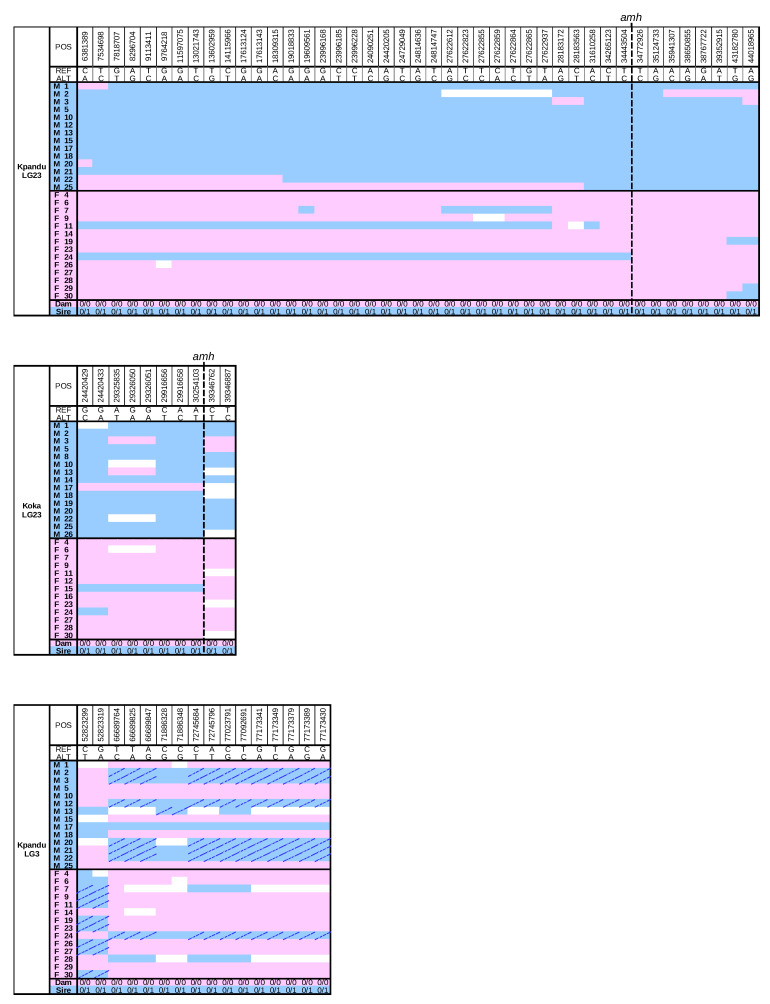
Genotypes of the offspring compared to their parents inferred from ddRAD FET significant sites following an XX/XY pattern. Pink cells indicate an individual having the same genotype as their mother at this site whereas blue cells indicate an individual having the same genotype as their father at this site (dashed cells indicate homozygous YY sites). White cells are missing data. Only sites having both parents and at least half males and females of the offspring sequenced were kept here. Those tested for a ZZ/ZW pattern are shown in Figure S3.

One advantage of using families for the ddRAD sequencing is that we can compare the SNPs of father and sons, and likewise mother and daughters, to determine whether the pattern of inheritance on LG23 corresponds to an XY or a ZW sex-determining system. We, therefore, focussed on sites for which both parents were sequenced. We found that most of the LG23 sites with a significant FET were consistent with an XX/XY pattern of inheritance (Figure 4).

In the Kpandu family, there was an increase in the concordance between genotype and phenotypic sex as we approached the *amh* gene on LG23 (Figure 4). The *amh* gene, which remains the strongest candidate for sex determination [43], is centred at 34.501 Mb. At the closest flanking SNPs (34,772,926 bp and 35,124,733 bp) all the offspring have genotypes that match their phenotypic sex, following an XY pattern of inheritance from their parents. The 7 SNP sites between 34,265,123 and 39,352,915 bp show a maximum of 3.6% (1/28) discordance between an XY genotype and a male phenotype in the offspring (Figure 4). Only one female shows a heterozygous genotype, at 34,443,504 bp, but she is also heterozygous for all previous sites on this chromosome. This pattern suggests a recombination breakpoint very close to the *amh* gene in this individual. At the 4 SNP sites after the amh gene (at 35,941,307, 38,650,855, 38,767,722 and 39,352,915 bp) there is a perfect match between genotype and phenotype, with the exception of one male presenting an XX genotype (Figure 4). Here again, we suspect a recombination breakpoint just after the *amh* gene in this individual.

The heterozygous signals are less obvious in the Koka fish due to the overall lower coverage and lower number of sites that passed the filters to build the genotypic table of parents and offspring. Nevertheless, there is a good correspondence between the genotypes of markers near *amh* and the sex of the offspring. The data are again consistent with an XX/XY pattern of inheritance (Figure 4). The genotypes immediately adjacent to the *amh* gene on LG23 follow an XX/XY pattern and are sufficient to predict the phenotypic sex of all but two individuals of the Kpandu and Koka families.

Although some markers on LG3 were statistically associated with phenotypic sex in the Kpandu family, the genotypes of individual markers were not sufficient to accurately predict the sex of every individual (Figure 4). Breakpoints between sites 52,823,319 and 66,689,764 may correspond to a region of increased recombination or a genomic rearrangement so that these markers are further apart than the reference genome suggests (Figure 4). However, the fact that females can be heterozygous or homozygous on either side of these breakpoints, and the same for males, indicates that this interval on LG3 probably does not contain a sex determiner. We also tested the Kpandu and Koka SNPs for a female ZW pattern but failed to detect a female heterozygous pattern (Figure S3). The statistical signal on LG3 may be an artifact arising from the highly repetitive sequences on this chromosome. 

### 3.4. Whole-Genome Sequencing Analysis

#### 3.4.1. B Chromosome Presence Creates Noise in the WGS Data

**Figure 5:**
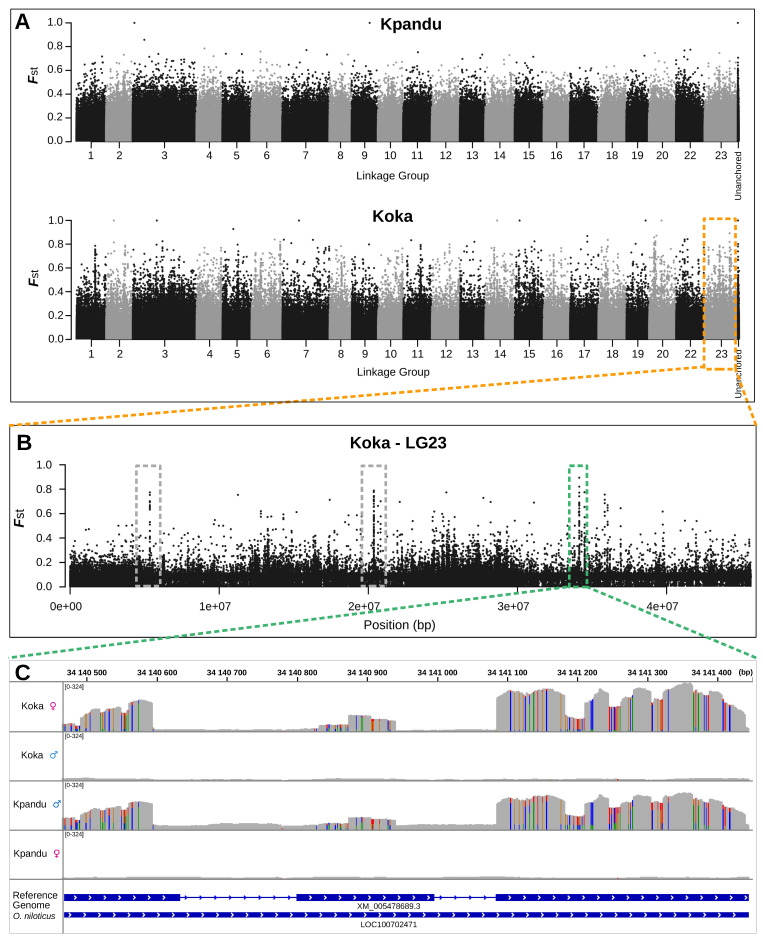
SNPs associated with phenotypic sex obtained from the WGS. (**A**) Manhattan plots of F_ST_ for Kpandu and Koka populations obtained with Sex SNP Finder. High F_ST_ peaks are observed on every LG meaning there is a signal associated to sex on all chromosomes; (**B**) Close up of F_ST_ on LG23 for Koka showing three high Fst regions in grey and green boxes; (**C**) Integrative Genomics Viewer (IGV) plot of sequence coverage at ~34.14 Mb on LG23 corresponding to the green box above for both sexes of the two wild populations. High F_ST_ values in this region are due to a very high sequence coverage (>10 times the mean coverage) likely corresponding to repetitive sequences on the B chromosome. SNPs are represented by colour traits with a bicolour trait being a polymorphic site, with the size of the colour band proportional to the nucleotide frequency. Frequencies of SNPs are conserved between Kpandu males and Koka females.

In the WGS data, every linkage group had many SNPs with unexpectedly high *F*_ST_ and significant Fisher’s Exact Test *p*-values between phenotypic males and females (Figure 5A). Taking a closer look at each of these high *F*_ST_ loci independently, we observed that this surprising signal was found in both populations and at the same positions, but it is clearer in the Koka population (Figure 5B). These high *F*_ST_ peaks correspond to regions with particularly high coverage and are typically observed in exons (Figure 5C). Similar high coverage polymorphic regions scattered in the genome have been attributed to the presence of highly repetitive B chromosomes in other African cichlids [26,63,64]. Thus, the presence of B chromosomes in our samples might explain the highly polymorphic regions of high sequence coverage observed in our study. At the species level, B chromosomes were not sex-specific since they were observed only in the Koka female pool and the Kpandu male pool. Consequently, the noise in *F_ST_* masks the polymorphism linked to the genetic sex-determinant. 

#### 3.4.2. Male-Specific Structural Variants in the Amh Region of LG23

**Figure 6:**
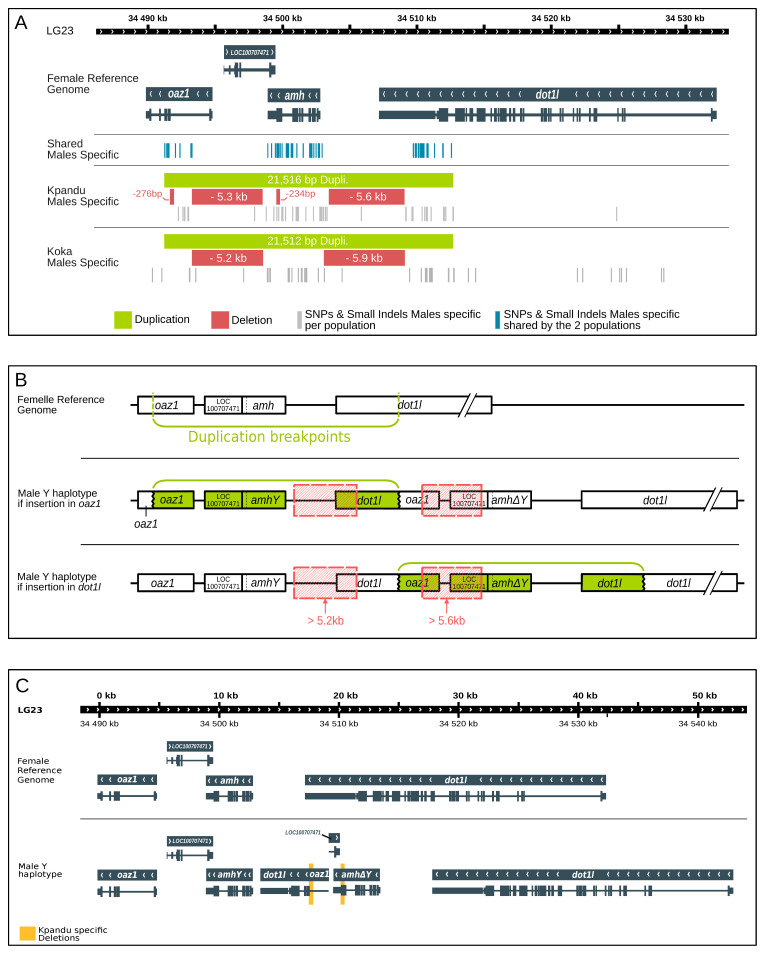
Schematic representation of the duplicated region of LG23 and its structural variants and polymorphic sites found with DELLY and FreeBayes. The tandem duplication includes the amh gene and part of the oaz1 and dot1l genes. The duplicated regions in each population (in green) are shown with the corresponding deletions and suggested size (in red). The shared male SNPs are shown in blue bars while the population male-specific SNPs are shown in light grey bars. (**A**) Schematic view of mapping results of the Y chromosome by WGS of the Kpandu and Koka males against the female XX reference genome. (**B**) Schematic representation of the two possible places of insertion of the tandem duplication in *oaz1* and *dot1l* genes. Both large deletions found with DELLY are likely to be placed in the *oaz1-dot1l* chimera as the genes are already truncated on the Y haplotype. (**C**) Schematic representation of the inferred Y haplotype of Kpandu and Koka males.

The whole-genome data allowed us to study in more depth the genome structure around the *amh* region in wild Kpandu and Koka males. The most striking structure we detected is a tandem duplication of 21.5kb located on the LG23 Y chromosome (Figure 6A) comparable to what Li et al. (2015) [43] found in a domesticated strain of Nile tilapia. Here we have found that the breakpoints on the X chromosome that led to the Y chromosome duplication are between two genes that flank *amh*. The ornithine decarboxylase antizyme 1 gene (*oaz1*) is found upstream of *amh* and the disruptor of telomeric silencing 1-like gene (*dot1l)*, also known as dot1-like histone lysine methyltransferase, is found downstream (Figure 6, Table 3). Both genes are transcribed on the same strand as the *amh* gene (antisense on the reference genome). The breakpoints we identified differ by only 4 bp between the Koka and Kpandu males. In the Koka males, the duplication spans the region from 34,491,225 bp and 34,512,737 bp, and in the Kpandu males spans the region from 34,491,225 bp to 34,512,741 bp on the XX female reference genome (Table 3). The confidence interval (CI) around the start and the end of the region that was duplicated is only 50 bp (Table 3) which allows us to say that the beginning of the duplication occurs after the third exon of the *oaz1* gene and ends before the last exon of *dot1l* (Figure 6A). The breakpoints take place within introns, and consequently, the duplication does not include the whole coding sequence for either of the two boundary genes (Figure 6B). Neither *oaz1* nor *dot1l* gene is fully duplicated on the Y haplotype. Instead, it creates a "chimera" comprised of a part of *dot1l* and *oaz1* genes, which occurs between the two copies of the *amh* gene on the Y chromosome. The insertion of this duplication on the Y chromosome could have taken place either upstream of the original Y structure within the *oaz1* gene or downstream within the *dot1l* gene (Figure 6B). It is important to note that Li et al. (2015) [43] positioned the *amhΔY* gene upstream of the *amhY* gene according to a sense strand. We, on the other hand, have defined the Y structure based on the new reference genome assembly where the reads in this region correspond to the anti-sense strand (Figure 6).

We also detected two large deletions of more than 5 kb that were common in males from both populations (Table 3 and Figure 6A,B). The first deletion was 5273 bp in length in Koka males and 5,233bp in length in Kpandu males (CI ± 390 bp and ± 150 bp, respectively) (Table 3). This male-specific variant encompasses a region between the first intron of *oaz1* and the *amh* gene according to the female XX reference genome (from 34,493,315 bp to 34,498,588/34,498,548 bp) (Figure 6). In the Y chromosome, the location of this large deletion depends on where the insertion of the duplicated sequences occurred. Either it starts in the complete copy of *oaz1* and ends close to the 5’UTR region of the *amhY* copy, or it starts in the truncated copy of *oaz1* and goes nearly up to the 5’UTR region of *amh∆Y* (Figure 6A). The first hypothesis implies that there would not be a complete *oaz1* copy on the Y chromosome. Thus, the second option where the deletion comprises an already truncated (and putatively non-functional) gene seems more plausible (Figure 6B).

The second common male deletion is 5,986 bp (CI ± 852 bp) in Koka, and 5,609 bp in Kpandu (CI ± 2 bp). It starts around 34,503,000 bp and ends at the same position (34,509,103 bp) in both populations (Figure 6A). This corresponds to the region downstream of the *amh* gene and upstream the last exon of the *dot1l* gene in the X reference genome (Figure 6A). Here again, there are two hypotheses for the position of this deletion on the Y chromosome. It could be located immediately after the complete copy of the *amh* gene and overlap the truncated *dot1l* copy, or it could be placed after the *amh∆Y* and overlap the complete copy of *dot1l* gene. For the same reasons as previously stated, the first option where the deletion comprises an already truncated gene seems more likely (Figure 6B).

From these data, we were able to describe the SD region of the Y haplotype compared to the X chromosome of the reference genome. After accounting for duplication and deletions sizes, the overall length of this region in males (starting from the first copy of *oaz1* to the last copy of *dot1l* genes) was estimated to be ~52 Kb whereas it is ~42 Kb in length in the female reference genome (Figure 6C).

#### 3.4.3. SNPs and Small Indels in the Amh Region in Males

A total of 60 SNPs and indels were found between males and females within the region encompassing *oaz1* to *dot1l* when mapping on the female reference genome (Table 4). However, all those SNPs and indels were described based on the female reference genome and using Illumina short reads. We are not able to confirm in which copies of genes (truncated or complete) these variants take place within the male’s duplication. A multiple nucleotide polymorphism (mnp) in the 3’UTR region, and 1 bp insertion in an intron within the *amh* gene, were observed in males of both populations. Five deletions are also shared in males from both wild populations in this region, which all occur in the 3’UTR part of the *dot1l* gene. A mnp is also detected in an intron of the *dot1l* gene. All other 52 shared polymorphisms are SNPs (Table 4), amongst which 9 are non-synonymous mutations: 2 in *oaz1*, 6 in *amh,* and 1 in the *dot1l* gene. Among the 52 SNPs found, the 3 in the *amh* gene and the 1 in *dot1l* are shared between the males of our 2 wild populations and the commercial stocks used in Cáceres et al. (2019) [44]. Among the 3 SNPs in *amh,* there is a T/C in exon 3, which corresponds to an A/G mutation in the transcript, changing the amino acid from threonine into alanine. The second SNP is a T/C occurring in exon 6, which corresponds to an A/G in the transcript, changing the amino acid from an asparagine into a serine.

#### 3.4.4. Population-Specific Sex Variants

We found other small deletions that were specific to the Kpandu population (Table 3). One of these deletions is 276 bp in length (CI ± 2 bp) and located in the first intron of the *oaz1* gene towards the limit of the second exon. There are two possibilities for the exact positioning of this deletion since it could be located either in the intact *oaz1* gene or in the truncated copy (Figure 6A,B). A second deletion of 234 bp in length (CI ± 3 bp) is located in exon 7 of the *amh* gene. It is placed in an exon, although it does not induce a frameshift. This means that at least one of the two copies of the *amh* located in the duplication is heavily modified (Figure 6C and Table 3) but from our short Illumina reads we are not able to say which (*amhY* or *amh∆Y*). Nevertheless, this 234 bp deletion confirms our PCR results when using the *amh∆Y*_-233_ marker (Figure 2) indicating that the *amh∆Y* gene is present in nearly all Kpandu males.

By using FreeBayes to look for small indels and SNPs, we observed 41 nucleotide polymorphisms and 6 small indels that are specific to Kpandu males (Table S4). All 6 non-synonymous mutations detected were within the *amh* gene, whereas indels were found in every gene in the *oaz1*-*dot1l* region, according to the female reference genome. In the Kpandu males, we found the 5bp insertion of exon 6 previously described by Eshel et al. (2014) [42] and Li et al., (2015) [43] which considered specific to the *amhΔY* gene. Here again, the sequencing data confirms our PCR results obtained with the *amhΔY*_+5_ marker (Figure 2). Other male-specific indels present in the Kpandu population are found in introns or the 3’UTR regions.

Koka males have 43 specific SNPs, 1 deletion and 1 insertion both in introns of the *amh* gene (Table S5). These include two non-synonymous mutations. A change of C/A at position 34,501,472 (corresponding to a G/T in exon 4 of the *amh* transcript), induces a change from arginine to serine. The second mutation is a C/A at position 34,521,953 is in an exon of the *dot1l* gene and causes a change from glycine to valine. The male-specific variants of Kpandu and Koka are distributed mainly over the same region as the shared males’ SNPs (Figure 6A). There are still some sites presenting male-specific variants that overlap with the deleted regions which means that they must appear in the intact copies of the genes. Finally, we found many more SNPs in Koka males than in Kpandu males for the *dot1l* region especially outside the duplication (Figure 6A, Table S5).

The WGS confirmed our PCR results (Figure 2) that Koka males do not have the 234 bp deletion found in Kpandu males, and which was reported to be specific to the *amh∆Y* copy in the Japanese strain [43]. Likewise, Koka males did not have the 5 bp insertion also considered to be *amh∆Y* specific according to the analyses of the Japanese strain [43], confirming our negative PCR amplifications (Figure 2).

## 4. Discussion

In the Nile tilapia, markers on LGs 1, 3, 20 and 23 have all been found associated with phenotypic sex in various domesticated strains [29,30,34,35,36,39,40,44,65]. To overcome possible artefacts due to the process of domestication, we have searched for the genetic basis of sex determination (SD) in wild populations of Nile tilapia and studied whether there are population differences. We chose two populations of Nile tilapia from geographically very distant locations, one from Kpandu (Lake Volta) in Ghana (West Africa) and the other from Lake Koka in the highlands of Ethiopia (East Africa). We opted to use a combination of two genomic approaches: double digest Restriction Associated DNA (ddRAD) sequencing on families with balanced sex-ratios and pooled Whole-Genome Sequencing (WGS) of wild-caught males and females. We found strong sex-associations with LG23 in both the Kpandu and Koka populations and a Kpandu-specific signal on LG3. We did not observe any association of sex with markers on LG1 or LG20.

### 4.1. Sex-Linked Signals Found on LG3

Our F1 ddRAD results showed a weak signal on LG3 that was found exclusively in Kpandu fish. LG3 is the largest chromosome pair of the *O. niloticus* karyotype [30]. It was presumed to be the sex chromosome after studies of chromosome pairing in the synaptonemal complex showed unpaired terminal ends of the largest chromosome in XY males [34]. Subsequent studies showed stronger linkage to LG1 [31,35,38,39] or LG23 [40,42,43,44]. In the sister species *O. aureus*, LG3 carries a female ZW determiner [30,66,67]. Several sex-patterned variants were found along a ~50 Mbp region in the long arm of LG3 in *O. aureus* when mapped onto the *O. niloticus* genome [67]. There is a very strong signal in the Kpandu family at 2 RAD loci located around 20Mb. One is located in a region of non-coding DNA. The other is found in the *fcgr2b* gene which codes for the receptor for Fc low-affinity II-b gamma immunoglobulin. Curiously, s*dy* which arises from a duplication of the immune system gene *irf9,* has become the master sex determinant in salmonid species [20]. The immune system showed higher sexual dimorphism for the *fcgr2a* gene, another gene of the “low-affinity immunoglobulin gamma Fc region receptor II” group in cichlids, a difference that was significantly greater in maternal mouth-brooders compared to biparental mouth-brooders [68]. The Nile tilapia is a maternal mouth-brooder, and we speculate that the difference in expression in *fcgr2b* might arise from sexual selection rather than being implicated in sex-determination.

The other sex-patterned SNPs on LG3 were found all along the “long-arm” of LG3. This linkage group is considered to have all the characteristics of an old sex chromosome in Nile tilapia, with signs of differentiation due to its large size and extensive heterochromatic regions with the accumulation of many repetitive sequences that may prevent recombination [51]. The SNPs we observe may result from the history of this pair of chromosomes and the recent accumulation of polymorphisms that do not follow sex-related patterns. This could explain why we did not find a definitive pattern of an XY or a ZW system for the LG3 SNP sites, even though a large number of highly differentiated SNPs were observed. Due to the high amount of repeated sequences in LG3 (54.7% repetitive vs. 37% genome-wide) only ~87 Mb of the chromosome could be assembled instead of the estimated ~130 Mb, and there might be incorrect anchoring to the *O. niloticus* genome [51]. We found on LG3 a very high proportion of immune-related genes and they were present in multiple copies. For example, 96 genes of the “Fc receptor” family are found on this linkage group, and among them 35 are “Low-affinity immunoglobulin gamma Fc region receptor II”. Consequently, the sex differences we observed may also result from mapping errors of reads and a lower quality of the assembly of this chromosome amplified by the random sampling of the ddRAD method.

### 4.2. Strong XY Patterns on LG23

Our family ddRAD results reveal that LG23 is an XY sex chromosome in both the Kpandu and Koka populations. LG23 has already been reported to be the sex chromosome in some domesticated Nile tilapia [41,43,44,65]. Among the significant sex-variants between 24 and 40 Mb on LG23, one is common to both populations: the anti-Müllerian hormone gene (*amh*). The *amh* gene has a primary role in the sex differentiation pathway of vertebrates including fish [69] and is implicated in germ cell proliferation in teleost fish (reviewed by Mullen et al. [70]). *Amhy* also appears to be the sex-determiner in several domesticated strains of Nile tilapia [40,41,42,43,44,65]. A recent study of a wild Lake Kou population (Burkina Faso) of Nile tilapia also showed that sex was linked to LG23 but the *amh* gene alone was not sufficient to explain all the sex-ratios observed [47].

We also looked at the other sex-significant sites found on LG23 and found a male-specific SNP for the *lingo3* gene in Kpandu fish. Variants have also been identified in *lingo3* in other Nile tilapia genomic data when searching for sex-determining regions [41,44]. The close linkage of the *lingo3* gene to the *amh* region probably explains the signal found in those previous studies and could be enough to explain the signal we found in the Kpandu individuals. In our two wild populations, we did not find any sex-signals on LG1 nor on LG20 which have both been found in the Manzala-Stirling strain [31,38,39].

Genotyping at the region with the most significant sex-pattern variants on LG23 was enough to perfectly predict the phenotypic sex of individuals in both populations. The Kpandu population belongs to the widespread Sudan-Sahelian cluster that separated from the Nile basin through multi paleo-geographic events [71]. The Koka population belonging to the Ethiopian Rift Valley showed large genetic divergence from the Sudan-Sahelian but also from the Nile basin from which it was isolated 12 to 8 thousand years ago [71]. The LG23 sex chromosome seems ancestral in *O. niloticus* since it appeared in the two wild populations studied here. This observation however contrasts with the apparent rapid evolution of the sex-determination in domesticated strains since at least three different LGs have been reported to be sex chromosomes. However, wild populations might segregate multiple sex determiners among which LG23 Y would be the most frequent and dominant. This may also explain why we found only LG23 in both the Koka and Kpandu families we selected. In wild Kou fish, for instance, sex was mainly associated with LG23 but it did not explain the phenotypic sex in 18% of the individuals, suggesting segregation of another sex-determiner [47]. This has already been shown for the Manzala-Stirling strain in which sex is strongly associated with LG1 and LG20 in some families [31,38] but it is also linked to LG23 in low frequency (Penman, unpublished data). Founding effects or domestication events have led to sex being determined exclusively by LG23 in the Manzala-Göttingen [65] and the Manzala-Tihange strains ([47], and this study) although they both originated from Manzala-Stirling breeders. Even stronger differences have been observed in the case of zebrafish where laboratory strains have lost the sex-chromosome found in wild populations [46]. Our study further highlights the importance of characterising the sex determiner in wild fish populations.

### 4.3. WGS Uncovers a Putative B Chromosome in O. niloticus

Blocks of high sequence coverage have been described previously in cichlid genomic data when B chromosomes were present, such as in *Astatotilapia latifasciata* [63,72], *Metriaclima lombardoi* from Lake Malawi [26], and the South American cichlid *Crenicichla lepidota* [73]. In these studies, high coverage regions were due to A chromosome fragments copied onto a B chromosome, where they are highly amplified and highly polymorphic. Because the reference assembly does not contain a B chromosome, the whole genome shotgun reads from all the different copies are mapped at the same position on the reference genome. The presence of these “B blocks” in our data, therefore, suggests the presence of a B chromosome in these two wild populations of Nile tilapia. In our study, the high coverage blocks mostly overlapped exons, which is a major difference from the B chromosomes previously described in other cichlids. This could be because the B chromosome is ancient, and the sequences corresponding to introns have diverged too much to map correctly onto the reference genome, unlike the exons that would be more conserved over time. Another hypothesis would be that genes accumulate on the B chromosome via an RNA intermediate, for instance through retrotransposition of mRNA, so the introns are not copied. Because the B blocks were found in Kpandu males and Koka females, they created strong differentiation between the sexes in each population, but these signals were independent of the true genetic sex-determining locus. It appears unlikely that the putative B chromosomes play a role as a female W sex-determiner such as in some Lake Malawi cichlids [27]. However, we are not able to determine the frequency of this B chromosome in our wild populations since we pooled the individual DNAs prior to sequencing. Even if the frequency is equal between sexes, if it is low in the wild, then a random sampling of individuals in our study could make it look like the B chromosome is sex-associated. B chromosomes have never before been described in Nile tilapia. Further studies will be needed to thoroughly characterize them in these populations. If B chromosomes are present in any of the domesticated strains, this can significantly impact genetic and genomic studies, from de novo reference assembly to identify sex-determining regions, as seen in this study. As such it would be beneficial to incorporate cytogenetic analysis as a control into the experimental design process.

### 4.4. Confirmation of the Tandem Duplication of the Amh Region on LG23

The Y chromosome *amhY* gene located on LG23 was shown to be necessary for maleness and is possibly the primary gene in a Japanese strain [43]. These authors also found the presence of a tandem *amh* duplication on the Y chromosome, with the existence of a truncated *amh∆Y* gene together with the *amhY* gene. Our work confirms the existence of this tandem duplication on the Y chromosome of LG23 in the two wild populations studied. We previously used four *amh* markers that distinguish the X and the Y-chromosomes that predicted the sexual phenotype with 100% certitude in the domesticated Manzala strain [47]. These markers also predicted sex correctly for 82% of the individuals in the wild Lake Kou population, while the remaining fish showed evidence of other epistatic loci [47]. We used these markers in both the Kpandu and Koka populations, but they were discriminative only in the Kpandu population. They allowed the identification of 3 XX males in the wild-caught fish, which were probably sex-reversed by high temperatures [48]. In contrast, only 2 markers corresponding to the *amh* gene amplified in the Koka population. Those corresponding to *amhΔY* did not amplify. These results initially suggested that the Koka males lacked the *amhΔY* gene or that the Koka sequences varied too much for our primers to amplify. However, our WGS allowed us to affirm with certainty the presence of this tandem duplication of the *amh* region with the presence of both *amhY* and *amh∆Y* genes in the Kpandu and Koka males.

### 4.5. Position of the Structural Variants in the Tandem Duplication of the Y Chromosome

We found two large deletions (>5 Kb) within the Y chromosome duplication that are shared by both wild populations. This could be evidence for a common origin of the male-specific *oaz1-dot1l* region between the two wild populations of Ethiopia and Ghana. One of these deletions overlaps *oaz1* and ends just before the last exon of the *amh*, whereas the second deletion occurs just before the first exon of the *amh* and overlaps the last exon of *dot1l*. There are two ways in which the deletion could overlap *oaz1* gene within the duplicated region: either it could overlap the intact copy of *oaz1*, or it could overlap the *dot1l-oaz1* chimera. The first scenario implies that there would not be a complete copy of *oaz1* on the Y chromosome. Thus, we suggest that the second scenario seems more plausible because the deletion removes an already truncated (and putatively non-functional) copy of the gene. For the same reasons, we propose that the deletion overlapping the *dot1l* gene takes place within the *dot1l-oaz1* chimera on the Y haplotype. These conclusions are consistent with Li et al. (2015) [43] who describe a large deletion (5.6 Kb) in the *amhY* promoter. We suggest that this deletion is the one overlapping the truncated copy of the *dot1l* gene.

Amongst the various male-specific SNPs we found in the duplication region, 8 are shared with other laboratory and commercial strains previously described [43,44]. All the SNPs described in the Japanese strain and which we found in the *amh* gene of our wild populations, were reported by Li et al. (2015) [43] as being *amhΔY* specific. However, we did not find the missense SNP in exon 2 specific for the *amhY* gene possibly contributing to male sex determination in the Japanese strain [43], in neither the Kpandu nor the Koka males. Nonetheless, we detected 52 new male-specific mutations in the *oaz1* to *dot1l* region in our two natural populations. Among them, six are non-synonymous mutations found in the *amh* gene that are common to both Koka and Kpandu. Two of them are located in exon 7, changing a threonine to a serine (both hydroxyl-containing amino acids) and an alanine to a threonine substitution which implies a change from a non-polar hydrophobic to a polar hydrophilic amino acid. Another important missense mutation in both populations is present in exon 6 which changes a glutamic acid to lysine, a basic amino acid. The other *amh* missense located in exon 6 is also common with the Latin American strains studied by Cáceres et al. (2019) [44]. Their fish are all from commercial stocks originating from the GIFT strain, itself originating from crosses between five domesticated and five wild populations [33]. The threonine to alanine mutation of exon 6 (34,501,082 bp) is, therefore, a strong candidate for maleness as it is conserved across populations and strains and might induce a major difference between the *amhY* and the female *amh* gene in wild populations.

Because our study was conducted using Illumina short reads sequencing, we are not able to determine which copy of the genes (truncated or complete) in the Y duplication our SNPs are from. Thus, it is impossible to conclude whether the SNPs affect the presumed sex-determining *amhY* gene or if they occur in the supposedly truncated *amhΔY* copy. As shown in Figure 6A, none of the shared male-specific SNPs or indels (blue bars) overlap the two large deletions (red bars). This indicates that sex-specific SNPs are not found in the conserved copies of these regions. We suggest that the majority of the SNPs and small indels we found are on the truncated versions of the genes.

### 4.6. Polymorphism between Wild Populations in this SD Region

For the same reasons explained previously, it is likely that the majority of the population sex-specific variants are found in the truncated copies of genes within the duplication. Nevertheless, some SNPs specific to Koka males or Kpandu males overlap the large deletions. These variants must, therefore, be found in the intact copies of the tandem duplication. The Nile tilapia Kpandu (Ghana) and Koka (Ethiopia) populations have evolved divergently, partly because of their geographical isolation, but perhaps also because of their ecology [71]. Therefore, it is not surprising to see population-specific polymorphisms.

None of the variants specific to Koka males have previously been reported in the literature. In contrast, we found several genomic variants (small and large indels) in Kpandu males that have been identified in the past in domesticated strains. This is the case for the 5 bp insertion in exon 6 and the 234 bp (CI ± 3 bp) deletion in exon 7 of the *amh* gene which are specificities of the *amhΔY* according to Eshel et al. (2014) (who called it initially *amhy*) [42] and Li et al. (2015) [43]. The 5 bp insertion causes a pre-mature stop codon in *amhΔY*, which would indicate that it is a truncated copy. We determined that this 5 bp insertion both by using PCR markers and by WGS was absent in the Koka males. This raises the question of whether the *amhΔY* might be functional in Koka males.

Due to our mapping onto the latest reference Nile tilapia genome and by specifically looking at the *amh* region, we highlighted a high nucleotide diversity in both wild populations. We cannot define which versions of the *oaz1*, *dot1l* and *amh* genes the SNPs belong to, however, the number of variants between X and Y haplotypes is greater than what has previously been described in domesticated strains [40,43,44]. We also found that the SNPs are numerous in the sex-determining region in wild populations. We did not find amongst these the sex-specific SNP in exon 2 described by Li et al. (2015) [43], and therefore, maleness is not dependent on this SNP in either population. Because of this unexpected diversity in this sex-determining region, it is possible that previously designed *amh* markers might not be efficient in wild populations. This appears to be the case for the Koka population which requires the analyses of new markers to discriminate better the *amhY*, *amh∆Y* and *amhX* genes. This also raises the question of whether using the existing *amh* markers are sufficient to sex individuals of wild populations and could explain some of the results of Sissao et al. (2019) [47]. Designing primers close to structural variants might be an efficient way to sex both wild populations and domesticated fish, as our study suggests that the sex region of farmed GIFT strains is still closely related to the Kpandu population [40,44].

### 4.7. Limits of the Methods Used

Although ddRAD sequencing is a relevant method to quickly identify potential sex-signals, we showed it might be influenced by repetitive elements and “old sex-chromosome” sequences, producing confounding artefactual signals. The ability of ddRAD to detect a signal will vary depending on the region analysed and the position of the ddRAD loci. Problems associated with a low density of markers in “RAD” studies (incorrect estimation of nucleotide diversity across the genome, missing sites...) have been addressed previously (reviewed by Lowry et al. [74]) and are accentuated when using ddRAD methods which sequence even fewer loci across the genome. Our results highlighted the role of the *amh* gene as a major sex-determining gene, but by using ddRAD sequencing we might have missed smaller regions implicated in sex determination acting as minor genetic factors. A recent study on a wild population of Burkina Faso showed that *amh* genotyping predicted the phenotypic sex with only 82% success, suggesting the existence of other minor sex-determinants [47]. The existence of such minor genetic factors influencing sex determination has previously been shown [12]. Here we combined our ddRAD analyses with whole-genome sequencing (WGS). Pooling by sex is a good method to ensure high coverage with reduced costs. However, we found that the presence of B chromosomes with highly repeated sequences is an issue when using the sequencing of pooled wild individuals to look for sex-specific signals with WGS. One should make sure of the absence of such genomic structures, especially when studying wild populations as we found evidence for B chromosomes in two distant populations across Africa. Nevertheless, WGS did enable us to study in-depth the genome structure around the *amh* region in the genome of wild Kpandu and Koka males.

## 5. Conclusions

Our study is the first to search for the sex determiner in wild populations of Nile tilapia using genomic approaches. F1 family analyses with ddRAD sequencing indicated that the major Y male determinant was located in LG23, in both Lake Volta (Kpandu) in West Africa and Lake Koka from East Africa. While the sex-variants covered a region of about 16 Mb in Koka, it encompassed nearly the whole of LG23 in Kpandu. A weaker sex association was identified on LG3 only in the Kpandu population, although neither XY nor ZW signals were conclusive. We confirmed the existence of a tandem duplication of the sex determiner candidate *amh* gene, in the Y chromosome of both Kpandu and Koka males. The breakpoints of the duplicated region were shown to be between the *oaz1* and *dot1l* genes. We found two common male deletions of ~5 kb, and the presence of both *amhY* and *amh∆Y* genes in both populations. We did not find the missense SNP in exon 2 which was thought to be important for maleness in a domesticated strain but found other male-specific variants. The *amh∆Y* gene in Koka males presented no structural variation, which raises the question of whether it is still a functional gene. Finally, WGS uncovered B blocks of high coverage suggesting for the first time in Nile tilapia the presence of B chromosomes in both populations that were not sex-related at the species level.

## Figures and Tables

**Table 1:** Fish samples were used in this study.

Approach	Population	Family	Sire	Dam	Sex-Ratio	Sampled Males	Sampled Females	Total Analyzed
ddRAD sequencing	Kpandu	Kp-20	KpM17	KpF32	49% (NS)	15	14	31
	Koka	Kk-6	KkM39	KkF4	55% (NS)	15	14	31
Whole Genome Sequencing	Kpandu					27	27	54
	Koka					20	14	34

Chi-square tests of observed vs. expected (1:1). NS for non-significant.

**Table 2:** Sex chromosomic LG23 *amh* markers analysed in the Kpandu and Koka wild-caught fish to predict the sexual genotype. The Manzala strain with known sex genotypes was used to validate the amplification.

Population or Strain		Phenotype	N Analyzed	Genotype	*amhX* Genotype	*amh∆Y* Genotype	*amhY* Genotype	Predicted Genotype
Domestic strain	Manzala	Females	1	XX	*amhX^+^*	*amh∆Y^−^*	*amhY^−^*	XX
		Males	1	XY	*amhX^+^*	*amh∆Y^+^*	*amhY^+^*	XY
			1	YY	*amhX^−^*	*amh∆Y^+^*	*amhY^+^*	YY
Wild populations	Kpandu	Females	27		*amhX^+^*	*amh∆Y^−^*	*amhY^−^*	XX
		Males	24		*amhX^+^*	*amh∆Y^+^*	*amhY^+^*	XY
			3		*amhX^+^*	*amh∆Y^−^*	*amhY^−^*	XX
	Koka	Females	14		*amhX^+^*	*amh∆Y^−^*	*amhY^−^*	XX
		Males	8		*amhX^+^*	*amh∆Y^−^*	*amhY^+^*	XY
			9		*amhX^+^*	*amh∆Y^−^*	*ND*	ND

**Table 3:** Structural variants located in the *oaz1-dot1l* region of LG23 in wild populations of Nile tilapia found with DELLY on whole-genome sequencing data.

Populations	Start Position	End Position	Confidence Interval around Start & End	Type	Size of Structural Variant	Shared between Populations
Koka	34491225	34512737	−50,50	Duplication	21512	Yes
	34493315	34498588	−390,390	Deletion	5273	Yes
	34503117	34509103	−852,852	Deletion	5986	Yes
Kpandu	34491225	34512741	−50,50	Duplication	21516	Yes
	34491681	34491957	−2,2	Deletion	276	No
	34493315	34498548	−150,150	Deletion	5233	Yes
	34499596	34499830	−3,3	Deletion	234	No
	34503494	34509103	−2,2	Deletion	5609	Yes

**Table 4:** SNPs and small indels found in the sex-determining *oaz1*-*dot1l* region on the Y haplotype between males and females of both populations.

Position	Reference	Alternative	Length	Type	Gene	Intron/Exon	Change of Amino Acid	Found in Literature
34491252	C	G	1	snp	*oaz1*	Exon		
34491407	A	C	1	snp	*oaz1*	Exon	Leu → Gly	
34491477	C	T	1	snp	*oaz1*	Intron		
34491574	T	C	1	snp	*oaz1*	Exon	Lys → Glu	Cáceres et al., 2019 [44]
34492052	A	G	1	snp	*oaz1*	Intron		
34492058	T	C	1	snp	*oaz1*	Intron		
34492381	A	G	1	snp	*oaz1*	Intron		
34493190	G	C	1	snp	*oaz1*	Intron		
34493277	A	G	1	snp	*oaz1*	Intron		
34498917	A	G	1	snp	LOC100707471	Intron		
34499201	CTAT	TTAC	4	complex	*amh*	3’ UTR		
34499475	A	G	1	snp	*amh*	3’ UTR		
34499597	C	T	1	snp	*amh*	Exon 7		
34499645	A	C	1	snp	*amh*	Exon 7		
34499675	A	G	1	snp	*amh*	Exon 7		
34499706	G	C	1	snp	*amh*	Exon 7	Thr → Ser	
34499810	A	G	1	snp	*amh*	Exon 7		
34499839	C	T	1	snp	*amh*	Exon 7	Ala → Thr	
34499987	C	T	1	snp	*amh*	Intron		
34499994	A	C	1	snp	*amh*	Intron		
34500307	C	T	1	snp	*amh*	Intron		
34500348	T	C	1	snp	*amh*	Intron		
34500364	G	C	1	snp	*amh*	Intron		
34500464	G	A	1	snp	*amh*	Intron		
34500668	C	A	1	snp	*amh*	Intron		
34500750	G	A	1	snp	*amh*	Intron		
34500773	C	T	1	snp	*amh*	Exon 6	Glu → Lys	
34501082	T	C	1	snp	*amh*	Exon 6	Thr → Ala	Cáceres et al., 2019 [44]
34501555	C	T	1	snp	*amh*	Intron		
34502034	T	C	1	snp	*amh*	Exon 3	Asn → Ser	Cáceres et al., 2019 [44]; Li et al., 2015 [43]
34502075	T	A	1	snp	*amh*	Exon 3		Li et al., 2015 [43]
34502169	ATG	AGTG	1	insertion	*amh*	Intron		
34502196	G	A	1	snp	*amh*	Intron		
34502353	A	C	1	snp	*amh*	Exon 2	Asp → Glu	Li et al., 2015 [43]
34502501	T	C	1	snp	*amh*	Intron		
34502686	T	C	1	snp	*amh*	5’ UTR		
34502748	T	C	1	snp	*amh*	5’ UTR		Cáceres et al., 2019 [44]
34502756	C	T	1	snp	*amh*	5’ UTR		Cáceres et al., 2019 [44]
34502954	G	A	1	snp	Non Coding DNA			
34509735	T	C	1	snp	*dot1l*	3’ UTR		
34509898	T	A	1	snp	*dot1l*	3’ UTR		
34509976	T	C	1	snp	*dot1l*	3’ UTR		
34510100	G	T	1	snp	*dot1l*	3’ UTR		
34510131	C	G	1	snp	*dot1l*	3’ UTR		
34510239	TTTAACT	TT	5	deletion	*dot1l*	3’ UTR		
34510250	A	C	1	snp	*dot1l*	3’ UTR		
34510260	A	G	1	snp	*dot1l*	3’ UTR		
34510309	A	G	1	snp	*dot1l*	3’ UTR		
34510392	A	G	1	snp	*dot1l*	3’ UTR		
34510428	GACA	GA	2	deletion	*dot1l*	3’ UTR		
34510495	T	G	1	snp	*dot1l*	3’ UTR		
34510529	C	T	1	snp	*dot1l*	3’ UTR		Cáceres et al., 2019 [44]
34510747	G	C	1	snp	*dot1l*	3’ UTR		
34510768	TGTGTGCG	TG	6	deletion	*dot1l*	3’ UTR		
34510842	CTTTTTTTTTTTTTTC	CTTTTTTTTTC, CTTTTTTTTC	5,6	deletion	*dot1l*	3’ UTR		
34511324	TAAATG	TAATG	1	deletion	*dot1l*	3’ UTR		
34511937	T	C	1	snp	*dot1l*	Exon		
34511950	A	T	1	snp	*dot1l*	Exon	Leu → His	
34511973	T	C	1	snp	*dot1l*	Exon		
34512585	ACAA	GCAG	4	complex	*dot1l*	Intron		

## Supplementary Materials

The following are available online at https://www.mdpi.com/2073-4425/11/9/1017/s1, Figure S1: Genotyping of wild-caught Kpandu fish of the AmhDY+5, AmhDY-233, AmhY-5608 and AmhX+36 markers; Figure S2: Genotyping of wild-caught Koka fish of the AmhDY+5, AmhDY-233, AmhY-5608 and AmhX+36 markers; Figure S3: Genotypes of the offspring compared to their parents inferred from ddRAD *FET* significant sites following a ZZ/ZW pattern; Table S1: Demultiplexing statistics for the ddRAD sequencing; Table S2: Details of the ddRAD FET-significant sites for LG23 found for both Kpandu and Koka populations; Table S3: Details of the ddRAD FET-significant sites for LG3 found for the Kpandu population; Table S4: SNPs and small indels found in the sex-determining *oaz1-dot1l* region on the Y haplotype-specific to the Kpandu population between males and females; Table S5: SNPs and small indels found in the sex-determining *oaz1-dot1l* region on the Y haplotype-specific to the Koka population between males and females.
