# Supplementary material for: Structure and Sequence of the Sex Determining Locus in Two Wild Populations of Nile Tilapia
Source: Genes (Basel). 2020 Aug 29;11(9):1017. doi: 10.3390/genes11091017 (PMC7563666; doi:10.3390/genes11091017)
Supplement: Supplementary file 1 [file genes-11-01017-s001.pdf]

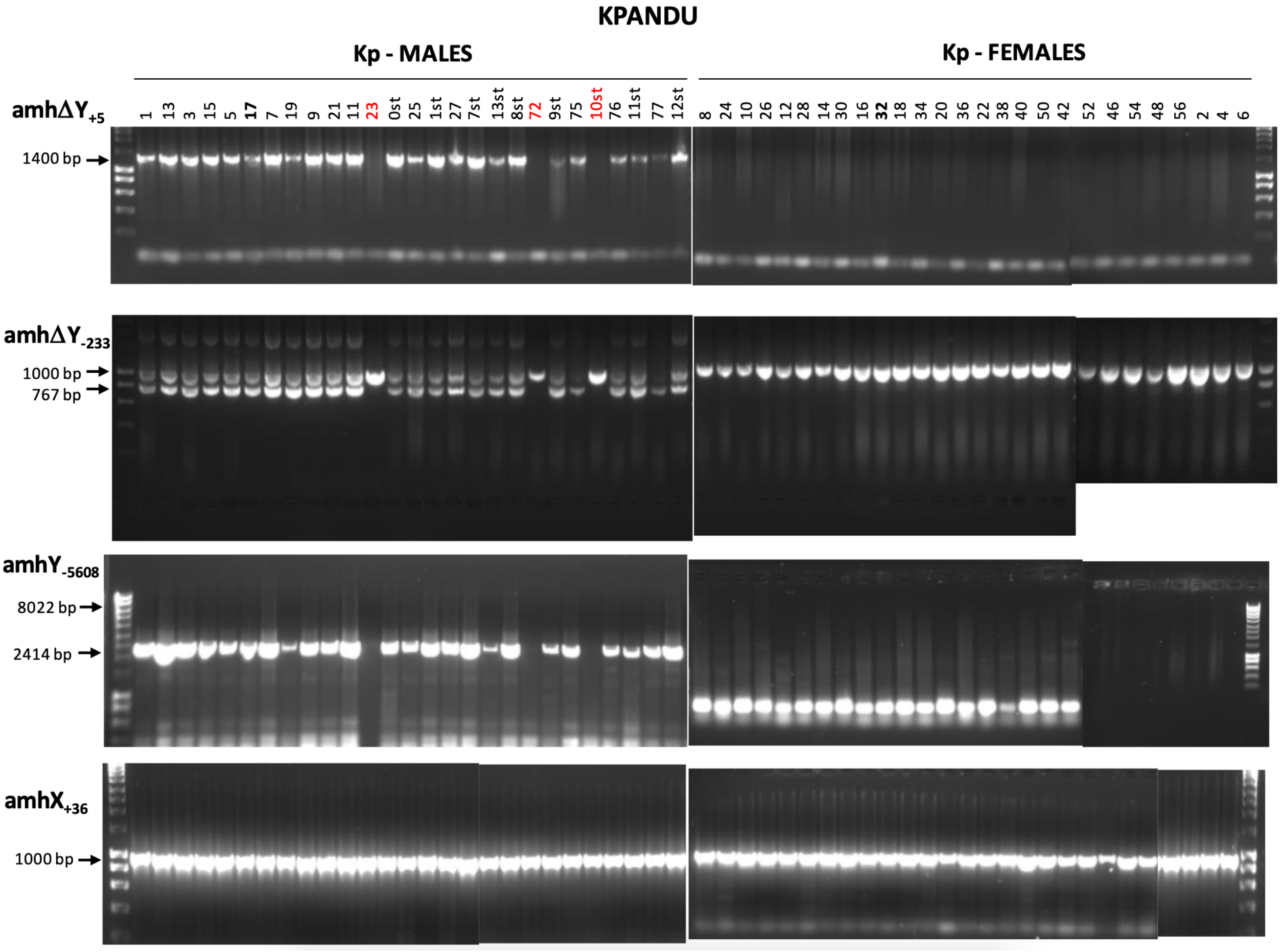

Figure S1: Genotyping of wild-caught Koka fish with the AmhDY+5, AmhDY- 233, AmhY-5608 and AmhX+36 markers

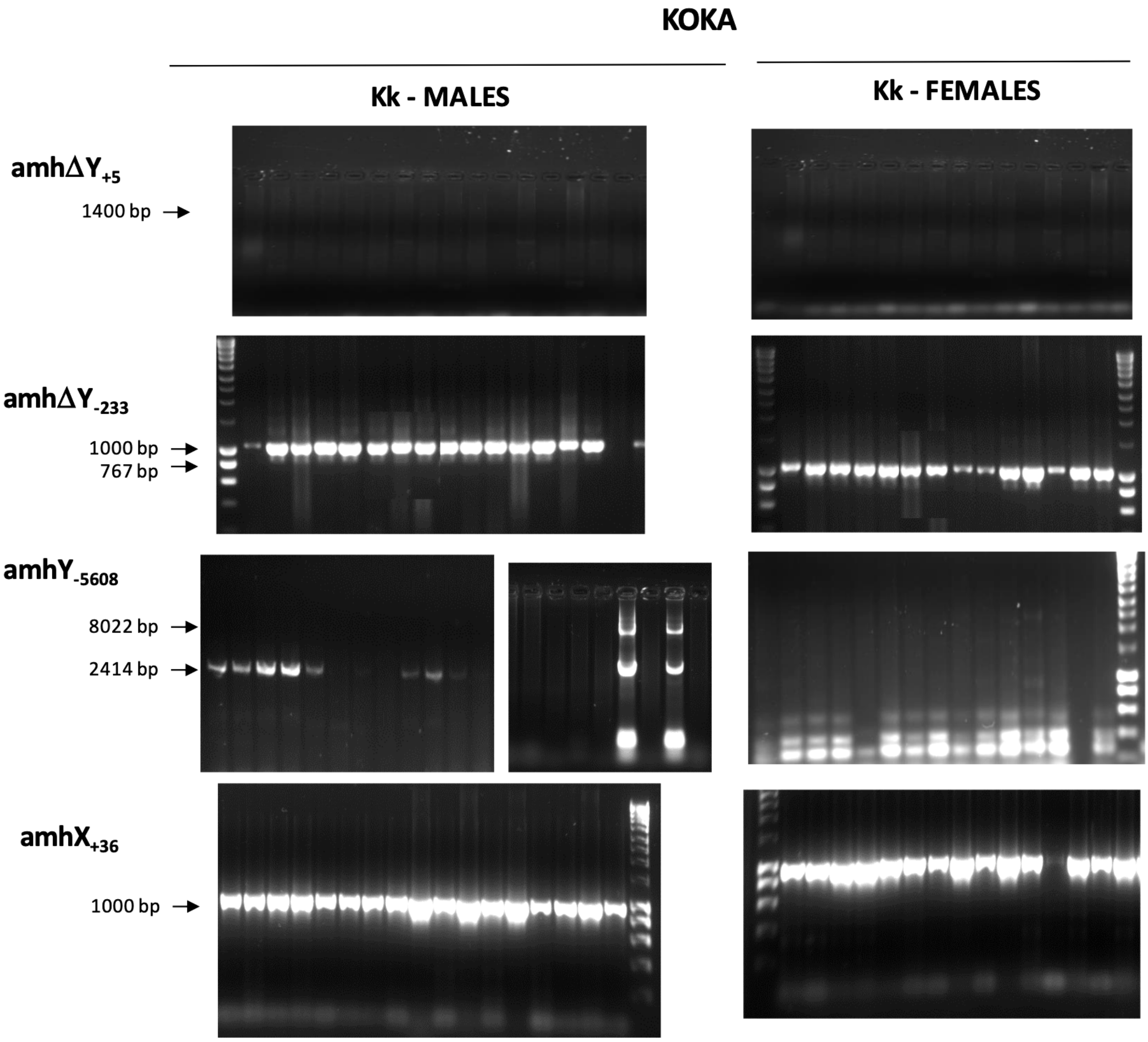

Figure S2: Genotyping of wild-caught Koka fish with the AmhDY+5, AmhDY- 233, AmhY-5608 and AmhX+36 markers

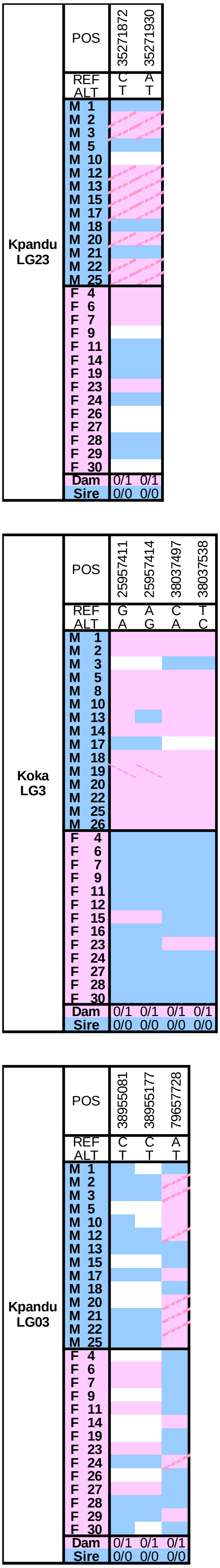

Figure S3. Genotypes of the offspring compared to their parents inferred from ddRAD FET significant sites following a ZZ/ZW pattern. Blue cells indicates an individual having the same genotype as it's father at this site whereas pink cells indicate an individual having the same genotype as it's mother (dashed cells indicates homozygous WW sites) at this site. White cells are missing data. Only sites having both parents and at least half males and females of the offspring sequenced were kept here

Table S1. Demultiplexing statistics for the ddRAD sequencing

Filename	Barcode	Total	No RadTag	Low Quality	Retained
Kk06_27-01	TCAGA-TAGCA	6519498	62393	10	6432161
Kk06_27-02	TGCAACA-AGCTGTC	6488002	42432	10	6421987
Kk06_27-03	GATCG-AGTCA	6130008	59742	12	6046134
Kk06_27-04	CGTATCA-TACGTGT	4786780	30645	11	4738595
Kk06_27-05	CATGA-GCATA	9155348	118757	17	9000160
Kk06_27-06	CACAGAC-GAGATGT	4304474	65547	8	4219379
Kk06_27-07	ATCGA-CGATC	6587634	72103	7	6492862
Kk06_27-08	ACTGCAC-CATCTGT	4087738	56399	2	4016341
Kk06_27-09	TCGAG-CTGGT	6728002	69386	11	6633318
Kk06_27-10	TCTCTCA-GTCAAGT	3997246	31884	7	3948143
Kk06_27-11	GTCAC-GAAGC	5398064	73342	3	5302251
Kk06_27-12	GTACACA-ATACGGT	5160480	37936	8	5104071
Kk06_27-13	GCATT-TAGCA	4329904	63290	8	4251195
Kk06_27-14	CTCTTCA-AGCTGTC	5150804	63152	12	5066501
Kk06_27-15	CGATA-AGTCA	6795198	67075	10	6702247
Kk06_27-16	CTAGGAC-TACGTGT	4458124	25108	8	4416516
Kk06_27-17	ACGTA-GCATA	8356696	150957	20	8170910
Kk06_27-18	CAGTCAC-GAGATGT	4402590	52193	9	4333027
Kk06_27-19	AGAGT-CGATC	4436860	57079	7	4363275
Kk06_27-20	GCTAACA-CATCTGT	6733466	59885	10	6650901
Kk06_27-21	ATGCT-CTGGT	45828	30344	0	15428
Kk06_27-22	ACACGAG-GTCAAGT	2569408	24098	3	2534472
Kk06_27-23	GACTA-GAAGC	3222708	63755	2	3145377
Kk06_27-24	AGGACAC-ATACGGT	3725288	58448	3	3652006
Kk06_27-25	TCAGA-AGCTGTC	2807592	36100	4	2760146
Kk06_27-26	TGCAACA-AGTCA	7424726	71086	9	7326480
Kk06_27-27	GATCG-TACGTGT	3747604	24415	3	3707727
Kk06_27-28	CGTATCA-GCATA	10693924	146273	20	10502658
Kk06_27-30	CATGA-GAGATGT	5827138	90528	4	5712289
KkGF04_27	GTACACA-AGCTGTC	4455130	44283	7	4392216
KkGM39_27	GCATT-AGTCA	3663614	43555	3	3606067
Kp20_27-01	CTCTTCA-TACGTGT	9118810	64960	19	9022280
Kp20_27-02	CGATA-GCATA	12392034	156916	21	12189103
Kp20_27-03	CTAGGAC-GAGATGT	7313068	69919	11	7218594
Kp20_27-04	ACGTA-CGATC	8325070	75518	20	8223412
Kp20_27-05	CAGTCAC-CATCTGT	7442942	72497	11	7344093
Kp20_27-06	AGAGT-CTGGT	5390846	113234	4	5259540
Kp20_27-07	GCTAACA-GTCAAGT	4501978	41902	9	4443363
Kp20_27-08	ATGCT-GAAGC	38146	28303	0	9777
Kp20_27-09	ACACGAG-ATACGGT	4740246	66828	2	4657183
Kp20_27-10	GACTA-TAGCA	6519380	92477	16	6403155
Kp20_27-11	AGGACAC-AGCTGTC	3317674	26002	9	3280807
Kp20_27-12	TCAGA-TACGTGT	5258990	48510	4	5192469
Kp20_27-13	TGCAACA-GCATA	10216096	71647	12	10106968
Kp20_27-14	GATCG-GAGATGT	6292344	36782	14	6232033
Kp20_27-15	CGTATCA-CGATC	7721200	71407	17	7624258
Kp20_27-17	CATGA-CATCTGT	11741310	81447	21	11625140
Kp20_27-18	CACAGAC-CTGGT	8255206	90916	12	8135409
Kp20_27-19	ATCGA-GTCAAGT	4667892	25809	5	4625598
Kp20_27-20	ACTGCAC-GAAGC	6783430	146471	12	6612036
Kp20_27-21	TCGAG-ATACGGT	7855194	42422	14	7787581
Kp20_27-22	TCTCTCA-TAGCA	7878918	111920	11	7741960
Kp20_27-23	GTCAC-AGCTGTC	7542616	44289	8	7472232
Kp20_27-24	GTACACA-AGTCA	8141530	65761	13	8047445
Kp20_27-25	GCATT-TACGTGT	5461980	34237	6	5408628
Kp20_27-26	CTCTTCA-GCATA	15576420	125831	22	15395325
Kp20_27-27	CGATA-GAGATGT	4719534	39821	6	4662161
Kp20_27-28	CTAGGAC-CGATC	10168514	70911	13	10064095
Kp20_27-29	ACGTA-CATCTGT	8314098	50526	16	8237185
Kp20_27-30	CAGTCAC-CTGGT	7810766	115129	8	7669778
KpGF32_27	AGAGT-GTCAAGT	2776938	36878	4	2729496
KpM17_27	GCTAACA-GAAGC	6458654	42653	7	6393873
Neg Control	ATGCT-ATACGGT	4408	2433	0	1965

Table S2. Details of ddRAD FET-significant sites for LG23 found for both Kpandu and Koka families

	BP	Fisher's Exact Test p-value	Amova Fst	Gene ID	Gene Start	Gene End	Product description
Kpandu LG23	3268204	5.42E-03	0.156	LOC109197015	3145390	4078398	ATP-dependent DNA helicase PIF1
	3268469	6.52E-03	0.148				
	5578395	2.36E-03	0.185	LOC100702162	5544973	5584661	multidrug resistance-associated protein 4
	6381389	5.65E-03	0.133	trak2	6373940	6389882	trafficking protein, kinesin binding 2
	6790753	9.93E-03	0.136	zranb3	6724257	6842613	zinc finger RANBP2-type containing 3
	7534698	2.60E-03	0.155	LOC100689728	7501806	7649676	gamma-aminobutyric acid receptor subunit gamma-3
	7818707	1.15E-03	0.179	Non Coding			
	8296704	1.15E-03	0.179				
	9113411	1.15E-03	0.179				
	9161445	2.61E-03	0.174				
	9161653	8.86E-03	0.134				
	9764218	2.25E-03	0.171				
	9982398	3.39E-03	0.190				
	11597075	1.15E-03	0.179				
	13021743	1.15E-03	0.179	LOC100699606	13020746	13022102	gamma-crystallin M3-like
	13602491	1.77E-03	0.203	Non Coding			
	13602504	4.45E-03	0.189				
	13602569	9.65E-03	0.165				
	13602575	1.95E-03	0.200				
	13602590	4.70E-03	0.171				
	13602591	1.95E-03	0.200				
	13602694	1.95E-03	0.200				
	13602701	9.19E-03	0.182				
	13602732	3.19E-03	0.196				
	13602959	1.15E-03	0.179				
	14115966	1.15E-03	0.179				
	17141915	8.95E-04	0.296				
	17613124	1.15E-03	0.179	als2	17587289	17614314	alsin Rho guanine nucleotide exchange factor
	17613143	1.15E-03	0.179				
	18309315	1.15E-03	0.179	Non Coding			
	19018833	9.10E-04	0.205				
	19031065	1.93E-03	0.221				
	19373150	7.34E-03	0.176				
	19609561	1.90E-03	0.166				
	19915347	2.61E-03	0.174				
	22988019	1.15E-03	0.232				
	22988087	1.15E-03	0.232				
	22988389	1.15E-03	0.232				
	23996168	9.10E-04	0.205				
	23996185	9.10E-04	0.205				
	23996228	9.10E-04	0.205				
	24090251	9.10E-04	0.205	LOC100690062	24067428	24119616	CLIP-associating protein 1-A
	24420205	9.10E-04	0.205	Non Coding			
	24729049	9.10E-04	0.205	agap1	24688557	24858705	arf-GAP with GTPase ANK repeat and PH domain-containing protein 1
	24814636	9.10E-04	0.205				
	24814747	9.10E-04	0.205				
	24932340	5.77E-03	0.156	LOC109196997	24910543	25412345	NLR family CARD domain-containing protein 3
	24932396	5.67E-03	0.156				
	27622612	2.88E-03	0.166	Non Coding			
	27622823	2.88E-03	0.166				
	27622855	3.73E-03	0.156				
	27622859	3.73E-03	0.156				
	27622864	2.88E-03	0.166				
	27622865	2.88E-03	0.166				
	27622937	2.88E-03	0.166				
	28006648	2.34E-04	0.276	LOC102077606	27999712	28030875	uncharacterized LOC102077606
	28183172	2.24E-04	0.224	LOC102077510	28169381	28190713	adhesion G-protein coupled receptor G7
	28183563	4.60E-04	0.216				
	28427026	3.64E-03	0.197	Non Coding			
	28720524	3.64E-03	0.156				
	28720537	3.64E-03	0.156	LOC100701816	28694684	28746200	NACHT LRR and PYD domains-containing protein 3
	28720563	3.64E-03	0.156				
	28720615	3.64E-03	0.156				
	28720616	3.64E-03	0.156				
	28720801	3.64E-03	0.156	LOC100701273	28706535	28721156	protein NLRC3-like
	28720868	3.64E-03	0.156				
	29326015	6.40E-04	0.255	mcf2l2	29307709	29404131	guanine nucleotide exchange factor DBS
	30529202	4.05E-04	0.221	LOC102076398	30446019	30539111	uncharacterized LOC102076398
	30529404	4.05E-04	0.221				
	30929265	7.83E-07	0.455				
	30929418	6.70E-05	0.329	LOC109196929	30927917	30936814	aldehyde dehydrogenase family 9 member A1-like
	30929419	3.19E-03	0.229				
	30944555	8.49E-04	0.214	LOC100707441	30940790	30950350	aldehyde dehydrogenase family 9 member A1
	31271536	4.05E-04	0.221				
	31271549	1.06E-04	0.260	evi5	31250049	31293025	ecotropic viral integration site 5
	31272084	1.06E-04	0.260				
	31610258	1.97E-04	0.231	Non Coding			
	33734321	8.90E-06	0.381	cyfip1	33706514	33746187	cytoplasmic FMR1 interacting protein 1
	33734328	8.90E-06	0.381				
	33907878	2.57E-05	0.309	celf5	33898573	34074566	CUGBP Elav-like family member 5
	34265123	3.30E-05	0.278	LOC100701388	34260519	34285917	zinc finger and BTB domain-containing protein 7A

Table S2. Continued

	BP	Fisher's Exact Test p-value	Amova Fst	Gene ID	Gene Start	Gene End	Product description
Kpandu LG23	34443504	3.30E-05	0.278	lingo3	34417222	34451018	leucine rich repeat and Ig domain containing 3
	34502301	2.82E-05	0.308				
	34502353	1.83E-05	0.297	amh	34498945	34502800	anti-Mullerian hormone
	34502501	1.83E-05	0.297				
	34772926	2.92E-06	0.333	tmem59l	34764172	34775066	transmembrane protein 59 like
	34774781	3.94E-05	0.286				
	35124733	2.92E-06	0.333	sema6b	35024697	35148164	semaphorin 6B
	35271872	4.63E-03	0.171	Non Coding			
	35271930	4.63E-03	0.171				
	35825054	1.76E-04	0.417	nwd1	35824755	35843686	NACHT and WD repeat domain containing 1
	35941307	8.38E-06	0.304	dbt	35940945	35947903	dihydrolipoamide branched chain transacylase E2
	35953054	2.89E-03	0.207	lrrc39	35948014	35953096	leucine rich repeat containing 39
	36092189	9.85E-03	0.176	grin3b	36077197	36130419	glutamate ionotropic receptor NMDA type subunit 3B
	36219951	1.83E-05	0.297	LOC102079807	36219513	36228399	leukemia NUP98 fusion partner 1
	36702603	3.54E-04	0.219	Non Coding			
	37501005	2.63E-03	0.180	lnx1	37471068	37507778	ligand of numb-protein X 1
	37989174	2.33E-03	0.161				
	38142119	7.70E-04	0.211				
	38142263	7.70E-04	0.211	Non Coding			
	38635419	3.81E-04	0.274				
	38650855	8.38E-06	0.304				
	38767722	8.38E-06	0.304	vamp4	38761399	38771204	vesicle associated membrane protein 4
	39345791	8.00E-03	0.143				
	39345873	8.00E-03	0.143	LOC106097024	39345054	39350322	cytochrome P450 2J6
	39345898	8.00E-03	0.143				
	39352915	8.38E-06	0.304	LOC102080408	39352504	39355741	cytochrome P450 2J6
	39495317	1.71E-03	0.259				
	39495320	2.90E-03	0.258				
	39495321	1.71E-03	0.259				
	39495351	1.71E-03	0.259	LOC100701255	39408394	39510887	cytochrome P450 2J2
	39495368	1.71E-03	0.259				
	39495382	1.71E-03	0.259				
	39495394	1.71E-03	0.259				
	39495409	2.90E-03	0.258				
	39687063	1.48E-04	0.282	ddr2	39674574	39709288	discoidin domain receptor tyrosine kinase 2
	39785274	4.25E-04	0.338	fsd1	39782941	39792956	fibronectin type III and SPRY domain containing 1
	39785344	2.58E-04	0.308				
	40703079	4.42E-04	0.304	LOC100691436	40679580	40708220	midnolin
	41258852	6.04E-03	0.178	LOC100710029	41229967	41308807	ELAV-like protein 4
	41863916	9.86E-03	0.121	safb2	41860641	41868251	scaffold attachment factor B2
	41864056	9.86E-03	0.121				
	43182780	9.10E-04	0.205	Non Coding			
	44018965	4.16E-03	0.142	LOC100706720	43894847	44022814	voltage-dependent R-type calcium channel subunit alpha-1E

	BP	Fisher's Exact Test p-value	Amova Fst	Gene ID	Gene Start	Gene End	Product description
Koka LG23	24256201	1.62E-03	0.240	Non Coding			
	24377090	1.00E-03	0.208				
	24377195	1.00E-03	0.208	LOC106098969	24373894	24379069	E3 ubiquitin-protein ligase TRIM21-like
	24377630	1.07E-03	0.208				
	24420429	9.76E-04	0.195	Non Coding			
	24420433	9.76E-04	0.195				
	24728973	3.19E-03	0.229	agap1	24688557	24858705	arf-GAP with GTPase, ANK repeat and PH domain-containing protein 17
	24729049	3.19E-03	0.229				
	25957411	9.76E-04	0.195	Non Coding			
	25957414	2.25E-03	0.171				
	29325835	2.38E-03	0.181				
	29326050	2.38E-03	0.181	mcf2l2	29307709	29404131	guanine nucleotide exchange factor DBS5
	29326051	2.38E-03	0.181				
	29595691	8.81E-03	0.131	Non Coding			
	29916656	1.18E-04	0.239	nlgn1	29685708	29978507	neuroligin 11
	29916658	1.18E-04	0.239				
	30030174	3.62E-03	1.000	naaladl2	29983730	30443760	N-acetylated alpha-linked acidic dipeptidase like 22
	30254103	1.18E-04	0.239				
	34502301	1.56E-03	0.188				
	34502339	1.62E-03	0.188	amh	34498945	34502800	anti-Mullerian hormone
	34502353	9.24E-03	0.133				
	34502501	1.56E-03	0.188				
	35097073	1.05E-06	0.762	sema6b	35024697	35148164	semaphorin 6B1
	35271920	1.13E-05	0.292	Non Coding			
	38037497	2.25E-03	0.171	impact	38027394	38052075	impact RWD domain protein
	38037538	2.25E-03	0.171				
	39346762	6.40E-04	0.255	LOC106097024	39345054	39350322	cytochrome P450 2J6
	39346887	6.40E-04	0.255				
	44145775	8.10E-05	0.493	LOC100702067	44143983	44228029	xenotropic and polytropic retrovirus receptor 1 homolog

Table S3. Details of ddRAD FET-significant sites for LG3 found for the Kpandu family

	BP	Fisher's Exact Test p-value	Amova Fst	Gene ID	Gene Start	Gene End	Product description
Kpandu LG3	20413224	7.68E-05	0.392				
	20413329	2.07E-04	0.361				
	20413333	2.07E-04	0.361				
	20413350	9.58E-04	0.322				
	20413371	7.68E-05	0.392				
	20413372	2.07E-04	0.361	Non Coding			
	20413385	2.07E-04	0.361				
	20413387	7.68E-05	0.392				
	20413395	7.68E-05	0.392				
	20413397	7.68E-05	0.392				
	20413407	7.68E-05	0.392				
	20766014	6.43E-03	0.357				
	20766054	6.43E-03	0.357				
	20766055	6.43E-03	0.357				
	20766056	6.43E-03	0.357	LOC109198185	20483081	21480470	low affinity immunoglobulin gamma Fc region receptor II-b
	20766057	6.43E-03	0.357				
	20766122	6.43E-03	0.357				
	20766236	6.43E-03	0.357				
	36091489	9.47E-03	0.190	LOC109198941	36064938	36268996	polymeric immunoglobulin receptor
	38181758	8.30E-03	0.154				
	38187085	4.82E-03	0.138	Non Coding			
	38955081	7.39E-03	0.183				
	38955177	9.53E-03	0.200				
	42505631	2.24E-03	0.381	LOC100695786	42240535	42871489	zinc finger protein 239
	42505666	9.66E-04	0.400	LOC102079722	42486440	42551726	uncharacterized LOC102079722
	46867058	1.80E-03	0.210				
	46867125	1.80E-03	0.210				
	46867130	1.80E-03	0.210				
	46867131	1.80E-03	0.210				
	46867155	1.80E-03	0.210				
	46867199	1.80E-03	0.210	LOC102075779	46859269	46890096	interferon-induced protein 44
	46867207	1.80E-03	0.210				
	46867229	1.80E-03	0.210				
	46867235	1.80E-03	0.210				
	46867244	1.80E-03	0.210				
	46867260	1.80E-03	0.210				
	46867275	1.80E-03	0.210				
	52823299	1.35E-03	0.218				
	52823319	1.16E-03	0.228	Non Coding			
	56398606	5.81E-03	0.132				
	56398607	5.81E-03	0.132				
	59781296	1.54E-03	0.314				
	59781378	1.54E-03	0.314				
	59781461	1.54E-03	0.314				
	59781466	1.54E-03	0.314	LOC102081234	59776051	59874428	interferon-induced protein with tetratricopeptide repeats 5
	59781493	1.54E-03	0.314				
	59781499	1.54E-03	0.314				
	59781506	1.54E-03	0.314				
	59781526	1.54E-03	0.314				
	61393476	4.82E-03	0.138	LOC100703578	60783576	61755773	nuclear factor 7, ovary
				LOC100708849	61376869	61661012	scavenger receptor cysteine-rich type 1 protein M130
	61443409	8.31E-04	0.325	LOC109196347	61441293	61444643	zinc finger BED domain-containing protein 1-like
	62886249	1.85E-03	0.174				
	62886306	1.85E-03	0.174	Non Coding			
	62925690	7.20E-03	0.169				
	64212311	3.97E-03	0.220	LOC112846310	64212138	64216441	selection and upkeep of intraepithelial T-cells protein 8-like
	65461749	2.14E-03	0.197	Non Coding			
	66629972	8.45E-03	0.130				
	66630036	3.46E-03	0.159				
	66630046	3.46E-03	0.159				
	66630065	2.52E-03	0.178	LOC109199604	66617589	66632971	uncharacterized LOC109199604
	66630117	3.46E-03	0.159				
	66630142	3.46E-03	0.159				
	66630215	3.46E-03	0.159				
	66689731	6.85E-03	0.159				
	66689764	1.23E-03	0.193	LOC102076984	66675533	66697030	immunoglobulin superfamily member 2
	66689825	3.22E-03	0.171				
	66689847	3.22E-03	0.171				
	67568787	1.62E-03	0.185				
	67799323	1.41E-03	0.268	Non Coding			
	67799376	1.91E-03	0.228				
	70202835	4.34E-03	0.179				
	70202841	4.34E-03	0.179				
	70202862	4.34E-03	0.179	LOC106096754	70191359	70259626	coxsackievirus and adenovirus receptor
	70202908	4.34E-03	0.179				
	70202916	4.34E-03	0.179				
	71886328	5.42E-03	0.156				
	71886348	5.05E-03	0.171				
	72745678	3.90E-03	0.158	Non Coding			
	72745684	3.90E-03	0.158				
	72745796	3.90E-03	0.158				

Table S3. Continued

	BP	Fisher's Exact Test p-value	Amova Fst	Gene ID	Gene Start	Gene End	Product description
Kpandu LG3	74381680	1.48E-03	0.188	LOC102082603	74377971	74409771	uncharacterized LOC102082603
	74381681	1.48E-03	0.188				
	74381692	1.48E-03	0.188				
	74381744	1.48E-03	0.188				
	74381746	1.48E-03	0.188				
	74381750	1.48E-03	0.188				
	74381754	1.48E-03	0.188				
	74381780	1.48E-03	0.188				
	74381798	1.48E-03	0.188				
	77023791	4.79E-03	0.155	Non Coding			
	77092691	4.79E-03	0.155				
	77173341	8.70E-04	0.218	cnga1	77170311	77177913	cyclic nucleotide gated channel alpha 1
	77173349	8.70E-04	0.218				
	77173379	8.70E-04	0.218				
	77173389	8.70E-04	0.218				
	77173430	8.70E-04	0.218				
	77201534	4.82E-03	0.138	nfxl1	77178317	77205828	nuclear transcription factor, X-box binding like 1
	77266911	9.03E-03	0.125	LOC109194306	77255116	77269156	sodium/hydrogen exchanger 9B2
	79657728	3.51E-03	0.148	LOC100699677	79653968	79662438	Beta-enolase

Table S4. SNPs and small indels found between males and females in the sex-determining *oaz1-dot1l* region on the Y haplotype specific to the Kpandu population

Position	Reference	Alternative	Length	Type	Gene	Intron / Exon	Change of amino acid	Found in literature
34492258	C	G	1	snp	*oaz1*	Intron		
34492617	G	A	1	snp	*oaz1*	Intron		
34492713	C	T	1	snp	*oaz1*	Intron		
34492746	ATAGTGTTTGTAGTGTTTGT	ATAGTGTTTGT	9	deletion	*oaz1*	Intron		
34492977	AAG	AACAG	2	insertion	*oaz1*	Intron		
34493031	A	G	1	snp	*oaz1*	Intron		
34497933	G	C	1	snp	LOC100707471	Intron		
34498818	A	C	1	snp	LOC100707471	Intron		
34499366	CAAAATATC	CAAATATC	1	deletion	LOC100707471 *amh*	5'UTR / 3'UTR		
34499592	G	A	1	snp	*amh*	Exon 7	Ala → Val	Eshel et al., 2014
34499740	T	C	1	snp	*amh*	Exon 7	Asn → Asp	
34499957	CA	TG,CG	2,1	mnp,snp	*amh*	Exon 7		Eshel et al., 2014
34500039	T	C	1	snp	*amh*	Intron		
34500045	C	G	1	snp	*amh*	Intron		
34500046	G	A	1	snp	*amh*	Intron		
34500246	A	C	1	snp	*amh*	Intron		
34500780	C	T	1	snp	*amh*	Exon 6		
34501028	C	T	1	snp	*amh*	Exon 6	Gly → Ser	
34501098	G	C	1	snp	*amh*	Exon 6		
34501125	CTC	CGACATTC	5	insertion	*amh*	Exon 6	Frame Shift	Li et al., 2015
34501180	G	A	1	snp	*amh*	Intron		
34501759	C	T	1	snp	*amh*	Intron		
34502301	A	G	1	snp	*amh*	Exon 2	Phe → Leu	
34502780	G	A	1	snp	*amh*	Intron		
34502864	G	C	1	snp	Non coding region			
34502934	CCG	TCA	3	complex	Non coding region			
34503031	C	T	1	snp	Non coding region			
34503062	G	C	1	snp	Non coding region			
34503220	A	G	1	snp	Non coding region			
34503326	CTC	CTCTTC	3	insertion	Non coding region			
34505875	A	C	1	snp	Non coding region			
34509196	T	C	1	snp	*dot1l*	3'UTR		
34509215	T	C	1	snp	*dot1l*	3'UTR		
34509624	A	T	1	snp	*dot1l*	3'UTR		
34509723	C	T	1	snp	*dot1l*	3'UTR		
34510382	GAAATATTT	GAAATAGCTACAAAATATTT	11	insertion	*dot1l*	3'UTR		
34510584	C	T	1	snp	*dot1l*	3'UTR		
34510698	C	T	1	snp	*dot1l*	3'UTR		
34510781	CATG	CGTC,CGTG	4,1	complex,snp	*dot1l*	3'UTR		
34511070	G	T	1	snp	*dot1l*	3'UTR		
34512003	CA	TG,CG	2,1	mnp,snp	*dot1l*	Exon		
34512023	A	G	1	snp	*dot1l*	Exon		
34512038	G	A	1	snp	*dot1l*	Exon		
34512159	A	G	1	snp	*dot1l*	Exon		
34512701	G	A	1	snp	*dot1l*	Intron		
34512721	CAG	TAT	3	complex	*dot1l*	Intron		
34524877	G	A	1	snp	*dot1l*	Intron		

Table S5. SNPs and small indels found between males and females in the sex-determining *oaz1-dot1l* region on the Y haplotype specific to the Koka population

Position	Reference	Alternative	Length	Type	Gene	Intron / Exon	Change of amino acid	Found in literature
34490339	G	A	1	snp	*oaz1*	Intron		
34491022	G	T	1	snp	*oaz1*	Intron		
34493089	G	C	1	snp	*oaz1*	Intron		
34493108	G	A	1	snp	*oaz1*	Intron		
34493560	T	C	1	snp	*oaz1*	Intron		
34497130	G	T	1	snp	LOC100707471	Intron		
34498890	G	T	1	snp	LOC100707471	Intron		
34499005	G	A	1	snp	LOC100707471	Intron		
34499106	G	A	1	snp	LOC100707471 *amh*	Exon / 3'UTR		
34500441	T	G	1	snp	*amh*	Intron		
34500513	G	T	1	snp	*amh*	Intron		
34500704	TTA	TA	1	deletion	*amh*	Intron		
34501083	G	A	1	snp	*amh*	Exon 6		
34501186	C	A	1	snp	*amh*	Intron		
34501405	G	A	1	snp	*amh*	Intron		
34501472	C	A	1	snp	*amh*	Exon 4	Arg → Ser	
34501667	G	T	1	snp	*amh*	Intron		
34501748	GAAAAAAAAAACAAGT	GAAAAAAAGAAACAAGT	1	insertion	*amh*	Intron		
34501797	T	A	1	snp	*amh*	Intron		
34502670	G	A	1	snp	*amh*	5'UTR		
34503139	G	T	1	snp	Non coding region			
34504441	C	A	1	snp	Non coding region			
34509462	C	A	1	snp	*dot1l*	3'UTR		
34510327	C	T	1	snp	*dot1l*	3'UTR		
34510652	T	C	1	snp	*dot1l*	3'UTR		
34510677	A	G	1	snp	*dot1l*	3'UTR		
34510759	C	G	1	snp	*dot1l*	3'UTR		
34510948	G	A	1	snp	*dot1l*	3'UTR		
34511044	A	G	1	snp	*dot1l*	3'UTR		
34511089	T	A	1	snp	*dot1l*	3'UTR		
34511147	T	A	1	snp	*dot1l*	3'UTR		
34512360	C	A	1	snp	*dot1l*	Intron		
34512391	T	A	1	snp	*dot1l*	Intron		
34512745	A	C	1	snp	*dot1l*	Intron		
34512777	T	G	1	snp	*dot1l*	Intron		
34513830	G	A	1	snp	*dot1l*	Intron		
34514369	C	T	1	snp	*dot1l*	Intron		
34521953	C	A	1	snp	*dot1l*	Exon	Gly → Val	
34522348	T	G	1	snp	*dot1l*	Intron		
34524490	C	A	1	snp	*dot1l*	Intron		
34525095	G	A	1	snp	*dot1l*	Intron		
34525239	G	A	1	snp	*dot1l*	Intron		
34525597	A	T	1	snp	*dot1l*	Intron		
34528214	G	A	1	snp	*dot1l*	Intron		
34528383	C	T	1	snp	*dot1l*	Intron		
